# Identification of phosphorylated proteins in erythrocytes infected by the human malaria parasite *Plasmodium falciparum*

**DOI:** 10.1186/1475-2875-8-105

**Published:** 2009-05-18

**Authors:** Yang Wu, Morag M Nelson, Andrew Quaile, Dong Xia, Jonathan M Wastling, Alister Craig

**Affiliations:** 1 Liverpool School of Tropical Medicine, Pembroke Place, Liverpool L3 5QA, UK; 2 Faculty of Veterinary Science, University of Liverpool, Crown Street, Liverpool L69 7ZJ, UK

## Abstract

**Background:**

Previous comparative proteomic analysis on *Plasmodium falciparum *isolates of different adhesion properties suggested that protein phosphorylation varies between isolates with different cytoadherence properties. But the extent and dynamic changes in phosphorylation have not been systematically studied. As a baseline for these future studies, this paper examined changes in the phosphoproteome of parasitized red blood cells (pRBC).

**Methods:**

Metabolic labelling with [^35^S] methionine on pRBC and 2D gel electrophoresis (2-DE) has previously been used to show the expression of parasite proteins and changes in protein iso-electric point (PI). 2-DE of different parasite strains was combined with immunoblotting using monoclonal antibodies specifically to phosphorylated serine/threonine and tyrosine, to obtain the phosphorylation profiles throughout the erythrocytic lifecycle. Affinity chromatography was used to purify/enrich phosphorylated proteins and these proteins from mature trophozoite stages which were identified using high-accuracy mass spectrometry and MASCOT search.

**Results:**

2D-immunoblots showed that *P. falciparum *infection greatly increased phosphorylation of a set of proteins in pRBC, the dominant size classes for phosphorylated tyrosine proteins were 95, 60, 50 and 30 kDa and for phosphorylated serine/threonine were 120, 95, 60, 50, 43, 40 and 30 kDa. The most abundant molecules from 2D-gel mapping of phosphorylated proteins in ItG infected RBCs were identified by MALDI-TOF. A proteomic overview of phosphorylated proteins in pRBC was achieved by using complementary phosphorylated protein enrichment techniques combined with nano-flow LC/MS/MS analysis and MASCOT MS/MS ions search with phosphorylation as variable modifications. The definite phosphoproteins of pRBC are reported and discussed.

**Conclusion:**

Protein phosphorylation is a major process in *P. falciparum-*parasitized erythrocytes. Preliminary screens identified 170 *P. falciparum *proteins and 77 human proteins as phosphorylated protein in pRBC, while only 48 human proteins were identified in the corresponding fractions from uninfected RBC. Refinement of the search to include significant ion scores indicating a specific phospho-peptide identified 21 *P. falciparum *proteins and 14 human proteins from pRBC, 13 host proteins were identified from normal RBC. The results achieved by complementary techniques consistently reflect a reliable proteomic overview of pRBC.

## Background

Phosphorylation-dephosphorylation is the major control mechanism for many cellular functions, including the regulation of cell division, protein synthesis and transcription [1]. Phosphorylation determines many functions of proteins (e.g. enzymes, microtubules, histones and transcription factors) and regulates signal transduction to control cellular responses to a particular stimulation. Phosphorylation often occurs on multiple distinct sites on a given protein [2] and this multi-layer control magnifies the final signal and promotes sensitive regulation. In a study by Ptacek *et al *using proteome chip technology to determine the *in vitro *substrates recognized by the majority of yeast protein kinases, 4,192 phosphorylation events involving 1,325 different proteins were identified. Approximately one third of all eukaryotic proteins are phosphorylated [3,4]. This represents a broad spectrum of different biochemical functions and cellular roles. Since many yeast proteins and pathways are conserved, these results provide insights into the mechanisms and roles of protein phosphorylation in many eukaryotes.

Protein phosphorylation has been shown to be an important event in malaria infection. For example, *Plasmodium falciparum *infection leads to a dramatic increase in the phosphorylation level of erythrocyte protein 4.1 which forms a tight complex with the mature parasite-infected erythrocyte surface antigens [5-7]. The 11 N-terminal amino acids of membrane-associated band 3 are critical for tyrosine phosphorylation and deletion of these amino acids greatly reduced the ability of parasite invasion [8]. By using [g-32P] ATP and [^32^P] orthophosphate labelling combined with 2-DE analysis, Suetterlin *et al *have identified 59 *P. falciparum *specific phospho-proteins with molecular weights between 15 and 192 kDa in pRBC [9], including two HSP70 heat shock proteins, Pf-hsp and Pf-grp [10]. *Plasmodium falciparum *infection causes the plasma membrane of erythrocytes to become increasingly permeable to a variety of physiologically relevant solutes via the induction of new permeation pathways (NPPs) which is also thought to involve phosphorylation [11]. Approximately 100 kinases have been identified in the *P. falciparum *genome, represent 1.1–1.6% of the protein-coding genes of malaria parasite [12,13] and there is great interest in them as potential targets for drugs based on chemical inhibitors [14]. Examples of *P. falciparum *phosphorylation activity include a calcium-dependent protein kinase that prefers phosphorylation of proteins of the host erythrocytic membrane but very few parasite proteins [15] and *P. falciparum *phosphatases such as protein phosphatase 1 (PP1), which is responsible for regulation of the phosphorylation status of the membrane protein PfSBP1 [16]. As inhibitors of protein kinases and phosphatases can interfere with parasite growth [17], it is suggested that studies in this area may contribute to the development of novel therapies for malaria.

Severe malaria is believed to be caused by adhesion of *P. falciparum *pRBC in the small blood vessels of major organs [18]. This processes involved in the reaction of several parasite ligands with endothelium receptors, mainly, PfEMP1 [19-22], ICAM-1 [23] and CD36 [24,25]. Protein phosphorylation has also been shown to relate to cell adherence events. In epithelial cells, tyrosine phosphorylation of cadherins and their associated proteins has major effects on the stability of adhesion junctions [26]. Alteration of tyrosine phosphorylation status is associated with the expression of tyrosine kinase receptor K-sam, which may cause the cytoplasmic distribution of cadherin-catenin molecules and loose cell-cell adhesion in undifferentiated-type gastric cancers [27]. In malaria, CD36 ectodomain phosphorylation at Thr^92 ^can regulate pRBC adhesion to CD36 and to human dermal microvascular endothelial cells (HDMECs) under flow conditions, and is regulated by a Src family kinase- and alkaline phosphatase dependent mechanism [28]. Lucchi *et al *demonstrated that pRBC cytoadherence to syncytiotrophoblast via chondroitin sulphate A (CSA) enhanced tyrosine phosphorylation of 93 kDa and 85 kDa proteins in these cells [29], but whether the phosphorylation was on protein of pRBC or BeWo cells has not been determined. Interestingly, a protein phosphatase inhibitor, levamisole reduced sequestration of *P. falciparum *trophozoites from malaria patients' peripheral blood, indicating a potential effect of dephosphorylation on *Plasmodium *pathogenesis [30]. It has been shown that parasite binding to endothelial cells activated MAPK signalling pathways and that the degree of phosphorylation was proportionate to the avidity of ICAM-1 binding [31]. Also related to the cytoadherence, phosphorylation of major erythrocyte proteins such as ankyrin and band 4.1 and 4.9 proteins can weaken the rigidity of the cytoskeleton thus reducing the binding efficiency of the infected erythrocyte [32].

It has been suggested that there is a correlation between parasite clones of different antigenic variants and cytoadherence, rosette formation and pathogenesis. These have led to studies on the diversity of these properties in *P. falciparum *isolates. One example is the line IT4/25/5 and its derivates [33]. ItG, A4 and C24 are representatives of parasite clones selected on C32 amelanotic melanoma cells (CD36) and HUVEC (ICAM-1), followed by sub-cloning. These three parasite clones have distinctive cytoadherence properties namely ItG binds strongly to ICAM-1 and to CD36, A4 binds strongly to CD36 and to ICAM-1, and C24 binds strongly to CD36 only. Another unrelated parasite line 3D7 is a low binding strain, which shows some binding to CD36 (although it can be selected for other binding specificities). It is clear that different parasite isolates have different binding phenotypes, but the molecules involved in adhesion and interaction with the host are still poorly understood. Moreover, very little is known about the phosphorylation status of the proteins involved in cytoadherence. Previous work on comparative proteomic analysis of parasite isolates with different adhesion properties found several changes in two-dimensional electrophoresis profiles in the protein isoelectric point (pI), consistent with protein phosphorylation as adding phosphoryl groups to a polypeptide chain supplies a negative charge to a protein [34], but the extent and dynamic changes of phosphorylation status have not been systematically studied. As a baseline for future studies, the changes in the phosphoproteome of different parasite lines at different stages of the asexual erythrocytic cycle were studied.

## Methods

### *Plasmodium *culture

*Plasmodium falciparum *isolates used in this study were: ItG [33,35] C24 [33], and 3D7 [36,37]. Parasites were cultured *in vitro *in group O^+ ^human erythrocytes using previously described conditions [38]. Briefly, parasites were cultured in RPMI 1640 medium (supplemented with 37.5 mM HEPES, 7 mM D-glucose, 6 mM NaOH, 25 μg ml^-1 ^gentamicin sulphate, 2 mM L-glutamine and 10% human serum) at a pH of 7.2 in a gas mixture of 96% nitrogen, 3% carbon dioxide and 1% oxygen. To minimize the effect of antigenic switching in culture, a batch of stabilates was prepared from post-selection cultures and used for no more than three weeks. To obtain specific lifecycle stages of *P. falciparum*, 5% sorbitol treatment or Plasmagel flotation was used. For this study, the trophozoites were obtained 24 to 32 hours after invasion and ring stages were obtained 6–10 hours after invasion.

### Metabolic labelling and fluorography

Parasites were synchronized with 5% sorbitol and left to grow to the trophozoite stage (28 hours after invasion). The parasitaemia was adjusted to 10% and cells washed with serum-free RPMI 1640 medium without methionine three times. *In vitro *metabolic labelling was carried out in RPMI medium lacking methionine, [^35^S] methionine was used at a concentration of 50 μCi ml^-1^. The culture was gassed and incubated at 37°C for four hours. The reaction was then stopped by centrifugation to remove radioisotopes. The labelled parasites were lysed as described previously and the Tris-insoluble pellet was subjected to 2D electrophoresis with pH 4–7 and 12.5% SDS PAGE. After electrophoresis, the gel was fixed and stained with Coomassie brilliant blue, immersed in Amplify (Amersham) for 30 min and dried for fluorography.

### Two-dimensional electrophoresis (2-DE) and immunoblotting

Two-dimensional electrophoresis was carried out according to Nirmalan *et al *[39]. Briefly, synchronized parasite culture was incubated in 0.05% saponin for 10 min in PBS on ice to lyse erythrocytes. The lysate was collected by centrifugation at 10,000 × *g *for 5 min and washed three times with 10 mM Tris-HCl pH 7.4 with 1 × protease inhibitor cocktail (Roche). The whole lysate was then solubilized in 2-DE rehydration buffer [8 M urea, 2 M thiourea, 2% CHAPS, 65 mM dithiothreitol (DTT), and 0.5% ampholyte pH 4–7 or 3–10]. The sample was vortexed and sonicated on ice 10 times for 5 seconds followed by centrifugation at 15,000 × *g *for 10 min. The supernatant was subjected to 2-DE and the isoelectric focusing (IEF) was run on precast Amersham 11 cm pH 3–10 immobiline Drystrip gels using IPG phor IEF Unit (Amersham). The running programme consists of 10 h for 30 V, 40 min for 200 V, 1 h for 500 V, 4 h for 2,000 V and finally 8 h for 8,000 V. The voltage was increased gradually until a total of 80,000 vh was reached. The focused strips were equilibrated in 10 ml equilibration solution (50 mM Tris-HCl, pH 6.8, 6 M urea, 30% glycerol, 2% SDS) with reducing agent of 1% DTT for 10 min, and 10 ml equilibration solution with 4.5% iodoacetamide for another 10 min. The strips were then washed twice briefly with 1× SDS gel running buffer and loaded on 10 or 12.5% SDS-PAGE gels for second dimension separation. The gels were run at constant current 40 mA in a Laemmli's buffer system until the dye front reached the bottom of the gel [40]. For 2D immunoblots, 2D gels were transferred electrophoretically to Hybond ECL nitrocellulose (Amersham GE Healthcare UK). The membranes were blocked with blocking buffer (Sigma UK) at room temperature for 30 min before being probed with the anti-phosphorylated tyrosine (diluted 1/2,000 in blocking buffer, Cell Signalling) or anti-phosphorylated serine/theronine antibody (diluted 1/2,000 in blocking buffer, Qiagen) at 4°C, overnight. The membrane was washed six times in Tris/saline/Tween (TST: 0.01 M Tris pH 8.5/0.15 M NaCl/0.1% Tween 20) for 10 minutes each time. Goat anti-mouse IgG (H+L) horseradish peroxidase conjugate (Nordic 1/2,000) was used to localize antibody-antigen complexes. The blot was developed for chemi-luminescence signals using Lumigen Solution (Amersham GE Healthcare, UK) and the results viewed by fluorography.

### Phosphatase treatment and purification of phosphorylated proteins

PhosProtein Purification Kit (Qiagen, Germany) was used to purify phosphorylated proteins from normal RBC and ItG infected pRBC according to the manufacturer's instructions. Briefly, 30 hours after invasion, pRBC were enriched by Plasmagel gel flotation from 250 ml culture (5 × 175 cm^2 ^culture flasks with 10% parasitaemia), to give a packed pRBC pellet of 2.5 ml. Normal RBCs were cultured under the same conditions for at least two days. The pRBC or RBCs were washed with culture medium three times followed by the hypotonic lysis of integrated pRBC or RBC. The lysates were washed extensively with 50 mM Tris-HCl, pH 7.5, and cold saline to remove haemoglobin, all these washing buffers contained 1× proteanase inhibitor (complete mini, Roche, Germany). For phosphatase treatment experiments, samples were resolubilized in protein lysis buffer (2 M urea, 2% CHAPS, 25 mM Tris-base, pH 7.5), half of each sample was treated with 10 units (in 10 μl) Shrimp Alkaline Phosphatase (SAP) or equal volume of 1 × enzyme storage buffer as a control, and incubated for 10–15 min at 37°C. Subsequent protein purification followed the manufacturer's instructions. Phosphorylated proteins eluted from the column were precipitated with 80% acetone/20% TCA in a ratio of 1:1 at -20°C overnight. The proteins were recovered by centrifugation at 15,000 × *g *for 20 min at 4°C, washed with 90% ice-cold acetone twice and re-suspended in SDS sample buffer, boiled for 5 min prior to 1D SDS-PAGE electrophoresis.

### Protein in gel digestion for MALDI and LC/MS/MS

Spot picking of phosphorylated proteins in 2D gels was guided by immunoblot images on duplicate gels. Enriched phosphorylated proteins were separated by 1D SDS-PAGE and excized into 34 slices. In gel digestion was performed as described previously [25], the excized protein was put into an Eppendorf Ultra Pure 1.5 ml centrifuge tube and cut into 1 mm^3 ^cubes. The gel slices were dehydrated by the addition of 100 μl of 50% (v/v) acetonitrile/water and incubated at room temperature for 10 minutes. The dehydrant was then replaced by 100 μl of ammonium bicarbonate (50 mM) and incubated again at room temperature for 10 min. These last two steps were then repeated. The gel slices were dehydrated again with 100 μl of 50% acetonitrile for 10 min and dehydrated slices were incubated in 10 – 20 μl of sequence grade trypsin (Promega) (10 μg/ml) for 18 hours at 37°C. The supernatant was retained and the gel pellet treated with 20 μl of 70% acetonitrile (v/v in water) for 60 minutes at room temperature. The supernatant from this step was then removed and pooled with the previous supernatant. The combined supernatant was dried in a rotational vacuum concentrator (RVC2–18), resuspended in 15 μl water, dried again and resuspended in 15 μl of 0.1% formic acid (in water).

### MALDI-TOF mass spectrometry

Samples from the in gel digestion were loaded in a sandwich manner with 1% cyano-4-hydroxycin-namic acid (Sigma) in 50% acetonitrile and 0.05% trifluoroacetic acid (TFA) onto a stainless steel target. High-resolution spectra were obtained using a Axima-CFR plus MALDI TOF instrument (Kratos Analytical, Manchester, UK) in reflectron mode. External calibration was performed using a mixed three point standard adjacent to the samples [25]. Acquisition and data processing are controlled by Launchpad software (Kratos Analytical, Manchester, UK).

Protein identification was performed by sending trypsin digested peptide masses to the *P. falciparum *and human databases of National Centre for Biotechnology Information (NCBI) using the MASCOT (Matrix Science) Peptide Mass Fingerprinting programme. Fixed carbamidomethyl modification and variable phosphorylation modifications were considered as parameters for the search programme. The mono-isotopic masses were used and the mass tolerance was set to 0.5 Da.

### Nanoflow LC/MS/MS analysis and database searching

The tryptic peptides were solubilized in 0.5% formic acid and were separated by nanoflow high-performance liquid chromatography on a C18 reverse phase column (Dionex Ultimate 3000) and elution was performed with a continuous linear gradient of 50% acetonitrile for 30 min. The eluates were analysed by online LC-MS/MS by using an LTQ ion-trap mass spectrometer (Thermo Finnigan). The resulting MS/MS spectra were submitted to TurboSequest Bioworks version 3.1 and the individual spectra was merged into an mgf file before sending to MASCOT (v2.2) for searching. Searching was against NCBI *P. falciparum *and human databases and was performed using fixed carbamidomethyl and variable phosphorylation modifications. Peptide tolerance was set 0.8 Da, MS/MS tolerance was set 0.5 Da. Phosphorylated protein identities were considered significant if the protein score was over the 95% confidence limit and at least one phosphorylated site was unambiguously identified when a phosphorylated residue existed (matched) y- or b- ions in the peak lists of the fragment ions [providing evidence of observed neutral loss of H_3_PO_4 _from the precursor or identified intact phosphorylated residues of Serine (pS), Threonine (pT) and Tyrosine (pY)]. If the protein score reached a significant level but the ion score of phosphorylated peptide was under the 95% confidence limit, these were referred to as potential-phosphorylated proteins.

## Results

**Figure 1:**
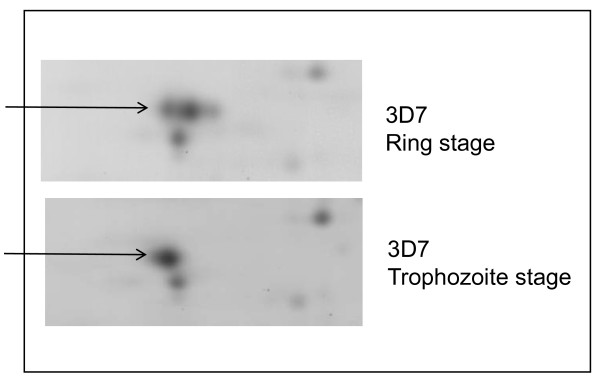
**Detail from a 2D gel image of proteins from *P. falciparum *3D7 line showing changes in pI between ring and trophozoite stages of pRBC**. The Tris-insoluble 3D7 pRBC pellets of ring stage and trophozoite stage were extracted and separated on 2D electrophoresis on pH 4–7 IEF strips followed by 12.5% SDS-PAGE. The gels were stained with Coomassie blue. The indicated protein was identified as human tropomycin by MALDI-TOF mass spectrometry.

**Figure 2:**
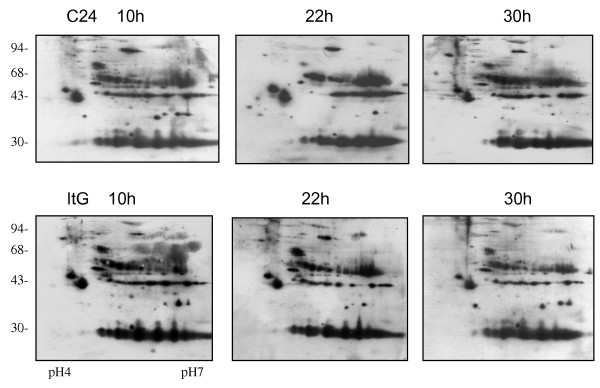
**Enhanced chemiluminescence images of immunoblots of phosphorylated serine/threonine proteins of different parasite lines at different time points on 2D gels**. C24 and ItG parasites were synchronized by double sorbitol lysis and proteins extracted at the time points indicated after parasite invasion. The Tris-insoluble pRBC pellets were extracted and separated on 2D electrophoresis on pH 4–7 IEF strips followed by 12.5% SDS-PAGE. The gels were transferred on to nitrocellulose paper, and probed with antibodies to phosphorylated serine/threonine.

**Figure 3:**
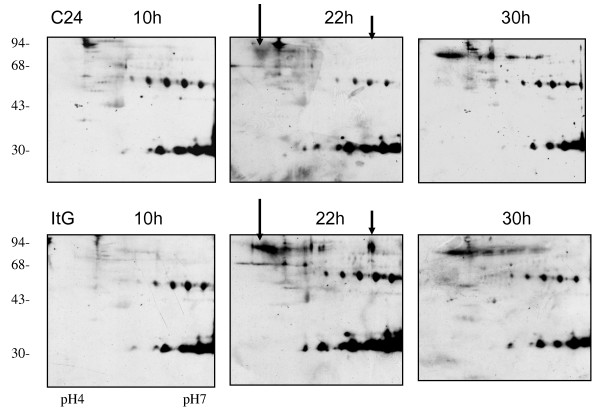
**Enhanced Chemiluminescence images of immunoblots of phosphorylated tyrosine proteins of different parasite lines at different time points on 2D gels**. C24 and ItG parasites were synchronized by double sorbitol lysis and proteins extracted at the time points indicated after parasite invasion. The Tris-insoluble pRBC pellets were extracted and separated on 2D electrophoresis on pH 4–7 IEF strips followed by 12.5% SDS-PAGE. The gels were transferred on to nitrocellulose paper, and probed with antibodies to phosphorylated tyrosine.

**Figure 4:**
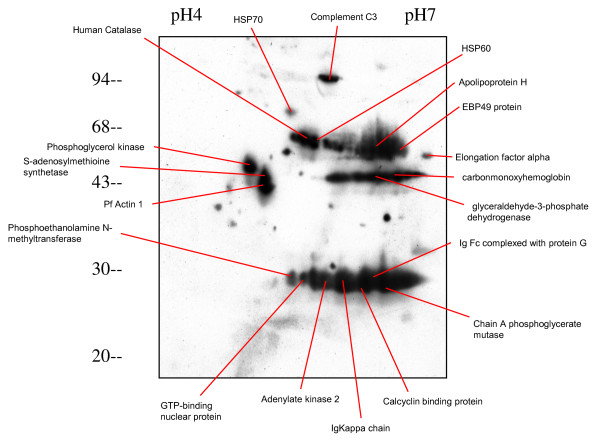
**Phospho-proteins separated by 2D gel electrophoresis of ItG trophozoite pRBC and positively stained by anti-phosphorylated serine/threonine at 22 hours after invasion**. The Tris-insoluble pRBC pellets were extracted and separated on 2-DE followed by immunoblotting. The duplicate gel stained with Coomassie blue was used to excise the corresponding protein spots and identify the proteins by MALDI-TOF or LC/MS/MS. All protein identities achieved significant protein scores in MASCOT search results.

As reported previously [34], using a sensitive *in vivo *labeling technique together with 2D electrophoresis revealed a number of changes in *P. falciparum *protein profiles from the pRBC of different parasite lines (Additional file 1). These changes were not limited to proteins from parasite source, but were also found in host proteins in the erythrocyte, varying during erythrocytic development of the malaria parasite e.g., PI changes of human tropomycin between ring and trophozoite stage (Figure 1). To map the phosphorylated proteins of pRBC in 2D gels, specific antibodies to phosphorylated amino acids (tyrosine and serine/threonine) were used to probe total pRBC lysates from a developmental series. As shown in Figures 2 and Figure 3, *P. falciparum *infection increased protein phosphorylation along with the developmental time course. There were approximately 50 protein spots that reacted positively with anti-serine/threonine antibodies and 4–5 groups of spots that extended horizontally like a family of different isoforms. The results showed that the dominant size classes for phosphorylated tyrosine proteins were 95, 60, 50 and 30 kDa and for phosphorylated serine/threonine were 120, 95, 60, 50, 43, 40 and 30 kDa. The major difference between ItG and C24 was the delayed appearance of a set of phosphorylated tyrosine proteins with molecular weight from 70 to 95 kDa, such that they appeared in ItG at 22 hours post-invasion and appeared in C24 at 30 hours. There were many low abundant proteins with pI changes in both strains, especially in phosphorylated serine/threonine, but it was difficult to view them in stained gels. The phosphorylation antibodies also recognized protein spots of 30, 45 and 95 kDa on non-infected erythrocytes (see Additional file 2) indicating that phospho-proteins are present in normal RBC as well (see below). The results indicated that parasite infection produced major phosphorylation changes in RBC irrespective of the stage. The majority of the proteins that reacted with anti-phosphorylated serine/threonine antibodies were identified by using MALDI-TOF and MASCOT search with phosphorylation modifications. Figure 4 indicates the proteins identified with significant scores and details of these proteins are listed in Table 1.

**Table 1:** Details of proteins identified from Figure 4

Protein No.	Accession Number	Protein name	Mol. wt	PI	Peptide identified^1^	Score
1.	(AAP72014)	Apolipoprotein H	37499	8.37	10	145

2.	NP_702487	Glyceraldehyde-3-phosphate dehydrogenase	37068	7.59	16	127

3.	P10988	Actin-1 (Plasmodium) P10988	42044	5.27	12	123

4.	NP_001107609	Erythrocyte membrane protein band 4.9 isoform	43118	8.87	15*	112

5.	XP_001352096	Phosphoglycerate kinase	45569	7.63	15	110

6.	AAV38387	Adenylate kinase 1	21735	8.73	11	108

7.	CAB45236	catalase	59947	6.90	17	105

8.	XP_001347854	GTP-binding nuclear protein ran/tc4	24974	7.72	23*	98

9.	C3HU	complement C3 precursor	188585	6.02	23*	95

10.	XP_001352093	s-adenosylmethionine synthetase, putative	45272	6.28	19*	91

11.	1K1K_B	Carbonmonoxyhemoglobin C	15970	7.98	12*	88

12.	NP_705453	Elonation factor 1 alpha	49156	9.12	18,	88

13.	BAB17688	Heat Shock Protein hsp70homologue pfhsp70-3	71945	5.90	30*	84

14.	AAD29608	Kappa 1 immunoglobulin light chain	26181	5.72	12*	78

15.	XP_001350775	Calcyclin binding protein, putative	30547	8.36	10	73

16.	1605217A	Ig gamma 1	25556	7.18	9	71

17.	XP_001347438	Pf-hsp60	62911	6.71	24*	66

18.	1XQ9_A	Chain A phosphoglycerate mutase	29891	8.31	23*	57

Note: 1. * = phosphorylated peptides identified

**Figure 5:**
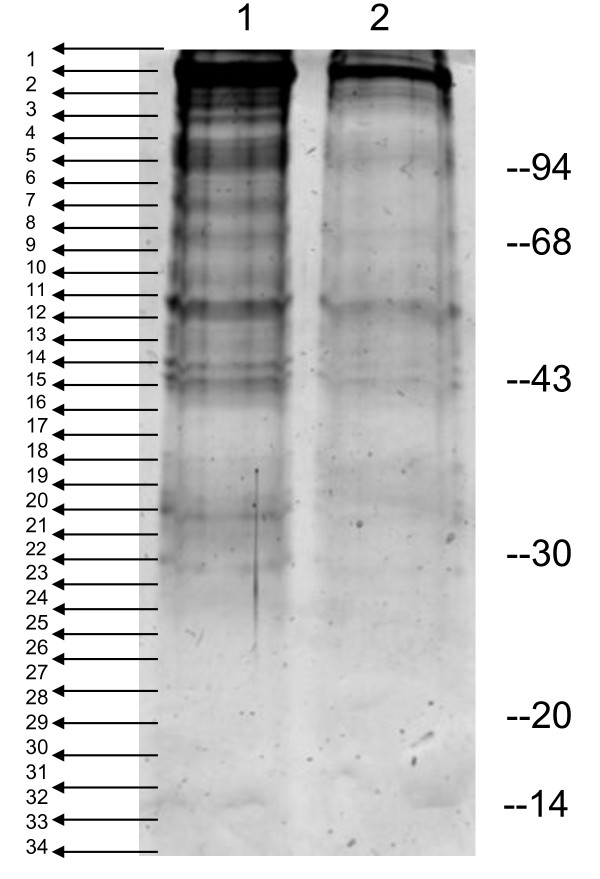
**1D gel image of proteins eluted from a phospho-protein purification column**. Lane 1, proteins with mock treatment, lane 2 proteins treated with SAP prior to purification/enrichment for phospho-proteins. The arrows indicate the cutting side and the numbers represent the bands excised and processed for LC/MS/MS analysis.

To improve the sensitivity of detection, phosphorylated proteins purified/enriched by affinity chromatography techniques were separated by 1D SDS-PAGE and identified by nano-flow LC/MS/MS. Figure 5 shows the gel images of the proteins treated with either SAP or mock buffer and then subjected to a PhosProtein Purification column (Qiagen). The phosphorylated proteins were eluted after extensive washing, precipitated and separated in 1-DE. The arrows indicate bands that were excised for LC/MS/MS analysis. When the whole pRBC lysate was pre-treated with phosphatase, the level and extent of proteins seen on the gel (lane 2) and therefore available for affinity purification was greatly reduced (NB: equal amounts of proteins were loaded on the affinity column). 34 bands were excised from lane 1 and the proteins identified represent a shotgun phosphoproteome of ItG trophozoites. All the peptide ions generated from LC/MS/MS were searched against NCBI databases using MASCOT search with and without phosphorylation modifications.

The proteins identified using searches including the phosphorylation modifications are listed in Additional file 3 (*P. falciparum *proteins from ItG-pRBC) and Additional file 4 (human erythrocyte proteins from ItG-pRBC). Additional file 5 contains the list of proteins identified from non-infected RBC. They include both confirmed and potential phosphorylated proteins. There were 170 *P. falciparum *proteins and 77 host proteins from purified ItG-pRBC phosphorylated protein pool, whereas in non-infected RBC, only 48 proteins were identified in the phosphorylated protein pool. To unambiguously identify phosphorylated peptides with phosphorylation sites, the phospho-peptides with significant ion score were further extracted to investigate their phosphorylation sites. These confirmed phosphorylated proteins are listed in Table 2. By using phosphorylation of serine/threonine and tyrosine as variable modifications, the selection criteria contains not only the total protein scores that reached a significant level, but also that protein hits included at least one significantly recognized phosphorylation site, giving further evidence in addition to their purification by affinity techniques for their phosphorylation status.

**Table 2:** The proteins possess significant ion score for phosphorylated peptides with defined phosphorylation sites

*Plasmodium falciparum *proteins from ItG-pRBCs:						
No*	Accession no	Protein name	Phosphorylated peptide sequences	MW*	Site*	Score*

PF1	PFB0100c	knob-associated His-rich protein	GASTTAGSTTGATTGANAVQSK	69791	T5, S8	103

PF2	PFE0870w	transcriptional regulator	FAYKSDEDDEGYNK	133456	S5	67

PF3	PF08_0137	hypothetical protein PF08_0137	KGSLGFDSFK	147208	S3	46

PF4	PF10_0159	Glycophorin-binding protein (GBP-130)	SVVTEEQKVESDSEK	90077	S11	37

PF6	MAL7P1.38	regulator of chromosome condensation protein	INEQSEGSNLPSEQNK	79931	S5	31

PF7	PFL0050c	hypothetical protein PFL0050c	AVPQNSGSNFDEFLDVK	77525	S8	32

PF5	PFI0265c	RhopH3	GLEFYKSSLK	104856	S7	33

PF8	PFD1165w	protein kinase, conserved	INDPYDYLKSITNQEER	75365	S10	61

PF9	PFE1485w	hypothetical protein	ETNESIYIK	225530	T2, Y7	45

PF10	PF14_0648	hypothetical protein	CDKEIYNLHIK	238305	Y6	36

PF11	PFE1600w	hypothetical protein	NYSTHENTYPTLK	62720	Y9	49

PF12	PF14_0434	hypothetical protein	FSVFDSDDNSEEEIENK	41909	S6, S10	42

			IIEVHDNIPSPIIK		S10	75

			DLNESPKNEPDIVYEEK		S5	59

PF13	PF13_0214	elongation factor 1-gamma	DDNNNNNNNDADNQHADLLSDDLAEK	50491	S20	48

PF14	MAL8P1.95	hypothetical protein	NLENNEDDETNVGR	37933	T10	44

PF15	PF07_0008	hypothetical protein	HYSLGGQPSSSGR	27713	S9	76

			NYSLGNLSTGTTSQGSTSSR		T11	76

			YNTSNLASSSTTESSVSGLNTNEAHV		S10	78

			NYSLGNLSTGTTSQGSTSSR		Y2 S16	58

			YNTSNLASSSTTESSVSGLNTNEAHV		T12	92

PF16	PFL1005c	chromodomain protein	NESPQWVEETNIR	30999	S3	55

			TGSDEEFEIGDILEIK		S3	98

PF17	MAL7P1.174	hypothetical protein	DGNDSSSEELDNTNVPTSKPK	37984	S5	63

PF18	PF13_0275	hypothetical protein	YGSGSHDHEEEVVEA	32877	S3	35

			SSSNSHVESNTFQNK		S3	47

PF19	XP_001351065.1	RESA-like protein	LNTIGNLFEGEK	36224	T3	54

PF20	Q8IKH8_PLAF7	ribosomal protein S3	TGLTSILPDNISVLEPK	24823	S12	46

PF21	PFD0375w	hypothetical protein	EYMNNILYDQNK	142869	Y2	36

Human proteins in ItG-pRBCs:						

Hu1	SPTB1	spectrin beta isoform b	KEELGELFAQVPSMGEEGGDADLSIEK	246894	S13	89

Hu2	M28880	Ankyrin	LGYISVTDVLK	207138	S5	62

Hu3	BAD92655	Ankyrin 1 isoform 4 variant	ITHSPTVSQVTER	208408	S4	55

Hu4	CAA41149	Erythrocyte alpha adducin	SPGSPVGEGTGSPPK	81377	S4	47

			AAVVTSPPPTTAPHK		S6	55

Hu5	AAH56881	ADD2 protein	TESVTSGPMSPEGSPSKSPSK	78576	S18	56

			SAGPQSQLLASVIAEKSR		S17	72

Hu6	AAH04261	Proline rich 7 protein	KIVTPFLSR	28986	T4	45

Hu7	MMHUE4	Erythrocyte membrane protein band 4.1	SLDGAAAVDSADR	95750	S1	65

Hu8	C3HU	Chain A, Complement Component C3	SGIPIVTSPYQIHFTKTPK	188585	T17	21

Hu9	Q05764	Beta-adducin	SPGSPVGEGTGSPPK	81129	S4	89

Hu10	CAH93400	Erythrocyte membrane protein band 4.9	SSSLPAYGR	45600	S3	38

Hu11	AAC50223	Dematin 52 kDa subunit	RGAEEEEEEEDDDSGEEMK	45726	S14	50

Hu12	NP_001284	Chloride ion current inducer protein I (Cln)	EPVADEEEEDSDDDVEPITEFR	26084	S11	55

			FEEESKEPVADEEEEDSDDDVEPITEFR		S17	60

Hu13	CAA40340	Glycophorin A	SPSDVKPLPSPDTDVPLSSVEIENPETSDQ	14784	S19, T27	43

Hu14	GFHUC	Glycophorin C	GTEFAESADAALQGDPALQDAGDSSR		S25	80

Human proteins from normal RBCs:						

RBC1	BAD92652	Spectrin, beta, erythrocytic	DASVAEAWLIAQEPYLASGDFGHTVDSVEK	268238	S27	62

			LSSSWESLQPEPSHPY		S4	27

RBC2	NP_000338	Spectrin	TSPVSLWSR	246468	S2	26

RBC3	NP_976217	Erythrocyte membrane protein band 4.1	VSLLDDTVYECVVEK	71955	Y9	58

			SLDGAAAVDSADR		S1	81

RBC4	AAL15446	Erythrocyte membrane protein 4.1N	TETMTVSSLAIRK	97405	S7 S8	23

RBC5	NP_001969	Erythrocyte membrane protein band 4.9	RGAEEEEEEEDDDSGEEMK	45514	S14	70

RBC6	CAA34611	Alt. ankyrin (variant 2.2)	ITHSPTVSQVTER	189011	S4	64

RBC7	NP_054909	Adducin 1 (alpha) isoform c	AAVVTSPPPTTAPHK	69985	S6	58

			SPGSPVGEGTGSPPK		S4	74

RBC8	AAH56881	ADD2 protein	TESVTSGPMSPEGSPSKSPSK	78576	S18	54

RBC9	NP_002427	Palmitoylated membrane protein 1	TAELSPFIVFIAPTDQGTQTEALQQLQK	52296	T20	53

RBC10	2GU8_A	Chain A, Discovery Of 2-Pyrimidyl-5-Amidothiophenes As Novel And Potent Inhibitors For Akt	TWDLCGTPEYLAPEIILSK	39232	T1	43

RBC12	1BUW_B	Chain B, Crystal Structure Of S-Nitroso-Nitrosyl Hemoglobin A	GTFATLSELHADK	15875	**S7**	31

RBC13	1DPF_A	Chain A, Crystal Structure Of A Mg-Free Form Of Rhoa Complexed With Gdp	DQFPAVYVPTVFENYVADIEVDGK	20169	Y7	23

No*: PF = Plasmodium protein; Hu = erythrocyte protein; MW* = protein molecular weight; site* = phosphorylated site identified by MASCOT; Score* = All phosphorylated peptide ion scores reached a significant level

GO slim software was used to classify these proteins into three categories: biological processes, cell components and molecular functions, producing three charts showing the percentage of proteins in specific categories (see Additional file 6). The height of each column indicates the numbers of proteins in each category, with different colours representing the proteins from the human host (blue) or the parasite (red).

## Discussion

The shotgun phosphoproteome of ItG-trophozoite pRBC revealed a total of 247 potential phosphorylated proteins with phosphorylated serine/threonine proteins (66.3%) more abundant than phosphorylated tyrosine proteins (33.7%). Most proteins identified by immune-blotting and 2D gel based MALDI-TOF techniques overlapped with proteins identified by affinity enrichment and LC/MS/MS analysis, supporting the specificity of techniques used. This is also indicated by the absence of abundant proteins in 2D gels (e.g. Pf-Enolase, Pf-Ornithine aminotransferase, human paraoxonase), indicated that the affinity chromatography technique selectively enriched a subset of proteins, ruling out general contamination of the samples, although this does not rule out a low-level of contamination.

Within the search algorithm variable modifications can be a powerful means of finding a match. MASCOT tests all possible arrangements of variable modifications to find the best match. For example, when Phosphorylation (Y) is selected, and a peptide contains 2 tyrosines, MASCOT will test for a match with the experimental data for that peptide containing 0, 1 or 2, tyrosine phosphorylation residues. Detection of phosphorylation is complicated because of site heterogeneity and the possibility of three fragmentation channels (intact fragments; neutral loss of HPO_3 _(80 Da); neutral loss of H_3_PO_4 _(98 Da). In MASCOT (v2.2), phosphorylated S, T and Y modification adds 80 Da, but whereas pY always stays intact in the spectrum, pS and pT can either stay intact or can lose 98 Da or occasionally 80 Da . In MS/MS ions search, confidence that a protein has been identified correctly comes largely from multiple matches to peptides from the same protein. For a suspected phospho-peptide, the calculated monoisotopic mass should include the phosphorylated residue(s) with individual ion scores that reach a significant level (Table 2). The potential phospho-protein 'hits' (Additional file 3, 4 and 5) were also included where MASCOT has identified enriched proteins with phosphorylation modifications, but their ion score of individual phospho-peptides failed to reach a significant score level.

Immunoblotting of various erythrocytic stages suggested that serine/threonine phosphorylation takes place early in the erythrocytic cycle as the phosphoproteome at 10 hours after invasion was very similar to that at 30 hours after invasion (Figure 2). However, if comparing immunoblots of Figure 2 and 3 with normal RBC (Additional file 2), the reaction was dramatically increased indicating that malaria parasite infection induced pRBC phosphorylation. 2D immunoblotting of phosphorylated tyrosine was relatively weaker compared to that of serine/threonine, consistent with our LC/MS/MS data and the general observation that phosphorylated serine/threonine proteins are abundant and phosphorylated tyrosine is relatively rare, for example in yeast, in human cells [41] and in bacterium [42]. There was an indication of changes on tyrosine phospho-proteins around 80–95 kDa in the trophozoite stage (Figure 3), between ICAM-1 binding (ItG) and non-binding (C24) parasite lines. However we failed to confirm their phosphorylation status due to the limited amount of proteins recovered from protein spots of 2D gels. In the gel-based study using MALDI-TOF, only two human proteins were significantly identified around 80–95 kDa, therefore we extracted the data from Additional files 3 and 4 which were obtained from phospho-protein enrichment by affinity chromatography and 1DE separation. There were many proteins significantly identified with molecular weights from 75–100 kDa (Table 3). 14 proteins were identified as tyrosine phosphorylated proteins by using LC/MS/MS, however it is not possible to say whether they are those seen in the 2D Immunoblot in Figure 3 although the molecular weight and positions in the gel would support this. Further more specific studies will be required to differentiate the phosphorylation status in parasite strains with different binding properties and to test if internal phosphorylation contributes to cytoadherence.

**Table 3:** Phosphoproteins from ItG trophozoites [75–100 kDa (see Fig. 5)]

Accession number	Gene name	Mol Wt	PI	Score	Phosphorylation Details*
PF10_0159	Glycophorin-binding protein 130	90077	5.08	779	ST2

PF08_0054	Heat shock 70 kDa protein (HSP74)	74754	5.51	444	ST4

BAG09363	Rhoptry complex polypeptide RhopH3	104798	6.25	324	ST3

MAL7P1.38	Regulator of chromosome condensation protein	79931	6.47	323	ST5, Y1

PFL0055c	Protein with DNAJ domain	108185	7.04	321	ST1, Y1

CAA28816	PFRESAR2 NID (ring-infected erythrocyte surface antigen precursor)	88816	4.92	181	ST1, Y1

Q8IFM1	Protein kinase	75365	9.08	153	ST8, Y1

PFF0250w	RNA binding protein, putative	86204	6.47	146	ST1

Q9GTW3	Glutamic acid-rich protein	80987	4.91	135	Y1

Q25730	Rhoptry associated protein-1	90423	6.69	121	ST20, Y1

Q8IJ23	Polyadenylate-binding protein	108656	8.35	110	Y1

PF11_0175	Heat shock protein 101	103038	9.17	103	ST1, Y1

Q25869	Heat-shock protein 86	86468	4.94	103	ST3

Q8I5H4	Polyadenylate-binding protein,	97455	8.96	94	ST11

Q8I3I6	Beta adaptin protein	102841	5.53	84	ST2

Q8I0V3	Chaperonin cpn60, mitochondrial	79898	4.93	76	ST2

E71608	ATP-dept. acyl-CoA synthetase (TP) PFB0695c	103277	8.83	63	ST8

PF10_0077	Eukaryotic translation initiation factor 3 subunit	84517	8.26	62	ST1

Q9NIH1	P101/acidic basic repeat antigen	86321	4.81	55	ST1

Q8I635	Hypothetical protein	77525	4.40	414	ST3, Y1

T18421	Hypothetical protein C0140c	89820	6.31	127	ST7, Y1

Q8IL87	Hypothetical protein	77412	9.07	90	ST2, Y1

Q8IL87	Hypothetical protein.-	77412	9.07	81	ST4

Q8I2G1	Hypothetical protein PFI1735c.-	82991	5.46	53	ST3

Host erythrocyte proteins					

Accession	Gene name	MW	PI	Score	Phospho-site

ADDB	Beta-adducin (Erythrocyte adducin subunit beta	80854	5.51	1207	ST7, Y1

MMHUE4	Erythrocyte membrane protein 4.1	95750	5.37	591	ST3

Q4VB86	Hypothetical protein (EPB41 protein)	83618	5.52	589	ST3

S18208	Rabphilin-3A-interacting protein	81260	5.67	456	ST7

AAD42222	AF156225 NID:	97567	5.22	382	ST3

ADDA	Alpha-adducin	81304	5.60	153	ST11, Y1

CAC18967	Sequence 3 from Patent WO0068693	85082	4.94	143	ST1 Y1

Q53FS6	TNF receptor-associated protein 1 variant	80326	8.05	72	ST3

I2C2	Eukaryotic translation initiation factor 2C 2	97991	9.34	46	ST1

* Phosphorylation details: The phosphorylated proteins were identified because they contained phosphorylated peptides. ST means serine/threonine, Y means tyrosine

The 30 kDa band seen with both anti-phospho tyrosine and serine/threonine antibodies shows some background, reacting with the secondary antibody only, but the intensity and extent of the reaction was greatly increased on the addition of the primary antibody. Moreover, it contains several known phospho-proteins, for example, calcyclin binding protein (gi23509063), adenylate kinase 2 (gi32394417), vesicle-associated membrane protein (gi23497447) and phosphoethanolamine N-methyltransferase (gi38018254). Some parasite erythrocyte membrane proteins such rifin (gi23613367) and stevor (gi23498318) have also seen when searching using MASCOT with phosphorylation modifications but they did not reach a significant level.

Many phosphorylated proteins seen in this study using affinity enrichment and LC/MS/MS showed very high MASCOT scores and frequently appeared in the search results suggesting high abundance or multiple family members. Since the proteins were extracted from Tris-insoluble pellets of pRBC, this pool includes proteins of outer and inner membranes, cytoskeleton and nuclear proteins. One of these proteins from *P. falciparum *is from the family of cytoadherence linked asexual proteins (CLAG), with five members of this family identified in phosphorylated protein pool namely CLAG 2, 8, 9 and RhopH1/Clag3.1, RhopH1/Clag3.2. These proteins are the products of *clag *gene family and have been proposed as being involved in cellular interactions [43,44]. Other abundant phosphorylated proteins of interest were DNAJ protein (PFE1605w), which is a co-chaperone of HSP70 in *E. coli *where it forms an operon with DnaK (HSP70) to regulate its ATPase activity [45]. In *P. falciparum *DnaJ is also referred to as RESA (in Additional file 3) and is predicted to be exported outside the parasite as it contains a PEXEL motif [46]. ISWI protein homologue (PFF1185w) is a chromatin-remodelling protein involved in gene expression and the maintenance of higher order chromatin structure [47] and possesses variable binding functions to DNA and proteins. There were a number of heat shock phospho-proteins, including: HSP101 (PF11_0175), HSP86 (PF07_0029), HSP78 (PFI0875w), HSP70 (PFI0875w), HSP74 (PF08_0054), HSP60 (PF10_0153) and other proteins with chaperone activities such as 14-3-3 protein homologue (MAL8P1.69) and Chaperonin CPN60 (PFL1545c). Molecular chaperones are a large protein family with roles in unfolding proteins for translocation, assembly and degradation. It is particularly interesting that most of these proteins are not only phosphorylated, but also exported [48,49], making them candidates for communication with the external environment. Known phosphorylated proteins found in this study were protein kinases and phophatases. However, the numbers and types of these enzymes identified were limited, which may reflect the situation *in vivo *that only small amount of regulatory molecules are present suggesting that the study of protein kinases or phosphatases in *P. falciparum *may need to use specific affinity techniques to produce suitable yields [50].

A basic level of phosphorylation was observed in non-infected erythrocytes with phospho-proteins including spectrin, ankyrin, adducin and erythrocyte membrane proteins 4.1 and 4.9. This kind of phosphorylation may be caused by the age of erythrocytes and *in vitro *culture, or may be a self-adjustment by the erythrocyte to maintain its shape and survival. By subtracting normal erythrocyte data from data obtained from ItG-pRBC, it was possible to distinguish some erythrocyte proteins significantly modified by phosphorylation upon invasion. One interesting protein identified as phosphorylated host protein in ItG-pRBC is NP_001284, chloride ion current inducer protein I (Cln), also called chloride channel regulatory protein. Malaria parasites infection produces high permeability of erythrocyte membrane to a large variety of solutes [51]. The altered permeability is presumed to be due to the activation of endogenous dormant channels and chloride channels are very important to channel activity [52]. Our data on the phosphorylation of a chloride channel protein in response to parasite invasion supports the possibility that this may contribute to NPPs [11]. Another interesting host protein is complement component 3 (C3) which we found as a phospho-protein specifically after *P. falciparum *infection. C3 is a component of the plasma but increases its deposition on red cells and binding to haemoglobin in children with severe malarial anaemia [53]. C3 is a critical regulator of innate immunity, implicated in the regulation of T cell-mediated responses [54]. It enhances the opsonization of immune complexes by phosphorylation due to increased binding to IgG [55]. There have been reports on the changes of complement system during malaria infection [56,57] and the contribution of complement in protective immunity in malaria [58], but little information about the phosphorylation states of complement components and their roles during infection.

## Conclusion

This study investigated the phosphorylation status of pRBC and dynamic changes of protein phosphorylation using Western-blots on 2D gels and/or phospho-protein enrichment coupled with high-accuracy mass spectrometry. The results have provided us with the basis of phosphorylation of RBC and pRBC for future research as well as identifying some interesting leads for further investigation of the role of protein phosphorylation in important parasite processes.

## Competing interests

The authors declare that they have no competing interests.

## Authors' contributions

YW: carried out the 2D-Immunoblot, MALDI, LC/MS/MS, data analysis and wrote the manuscript. MN: participated in the phosphatase treatment and phospho-protein enrichment. AQ: participated in protein identification using LC/MS/MS. DX: participated in LC/MS/MS analysis and data searching. JW: participated in the design of the study. AC: conceived of the study, and participated in its design, analysis of results analysis and writing the manuscript. All authors read and approved the final manuscript.

## Supplementary Material

Additional file 1 **PI changes of pRBC from different parasite lines**. Fluorography of proteins from trophozoite stage pRBC infected with 3D7 and ItG. At 20 hours after invasion, parasites were metabolically labelled with 50 μCi/ml [^35^S] methionine for 4 hours. Tris-insoluble pellets of pRBC were separated run on pH 4–7 IEF strips followed by 12% SDS-PAGE. Gels were stained with Coomassie blue, dried and exposed to X-ray film. Marked boxes show proteins with at least three fold changes between 3D7 and ItG. Enlarged images of corresponding boxes showing significant changes in the protein profiles. Arrows indicate the relative positions of the spots in different lines. Click here for file

Additional file 2 **Immunoblot of normal RBC and secondary antibody control**. Immunoblot of normal RBC separated by 2DE (using the same conditions as for Figures 2 &3) and probed with antibodies to phosphorylated serine/threonine (A) or tyrosine (B). Part C is an immunoblot of Tris-insoluble pellet of ItG-infected RBC probed with the secondary antibody only and developed by ECL. Click here for file

Additional file 3 ***Plasmodium falciparum *phosphorylated proteins from ItG infected erythrocytes**. Phosphorylated proteins purified/enriched by affinity chromatography techniques were separated by 1D SDS-PAGE and identified by nano-flow LC/MS/MS. Additional file 4 contains *P. falciparum *proteins from ItG-pRBC identified using searches including the phosphorylation modifications. Click here for file

Additional file 4 **Host phosphorylated proteins from ItG infected erythrocytes**. Phosphorylated proteins purified/enriched by affinity chromatography techniques were separated by 1D SDS-PAGE and identified by nano-flow LC/MS/MS. Additional file 5 contains human phosphorylated proteins from ItG-pRBC identified using searches including the phosphorylation modifications. Click here for file

Additional file 5 **Phosphorylated proteins of non-infected erythrocytes purified from phospho-affinity column**. Phosphorylated proteins purified/enriched by affinity chromatography techniques were separated by 1D SDS-PAGE and identified by nano-flow LC/MS/MS. Additional file 6 contains human phosphorylated proteins from normal RBC identified using searches including the phosphorylation modifications. Click here for file

Additional file 6 **Gene ontology analysis of serine/threonine and tyrosine phosphorylated proteins**. Gene ontology analysis of the serine/threonine and tyrosine phosphorylated proteins. A. Functional categories; B. Cellular components; C. Biological processes. The different colours indicate human (blue) or parasite (red) proteins. Click here for file
