# Supplementary figures and images for: Identification of phosphorylated proteins in erythrocytes infected by the human malaria parasite Plasmodium falciparum
Source: Malar J. 2009 May 18;8:105. doi: 10.1186/1475-2875-8-105 (PMC2696463; doi:10.1186/1475-2875-8-105)

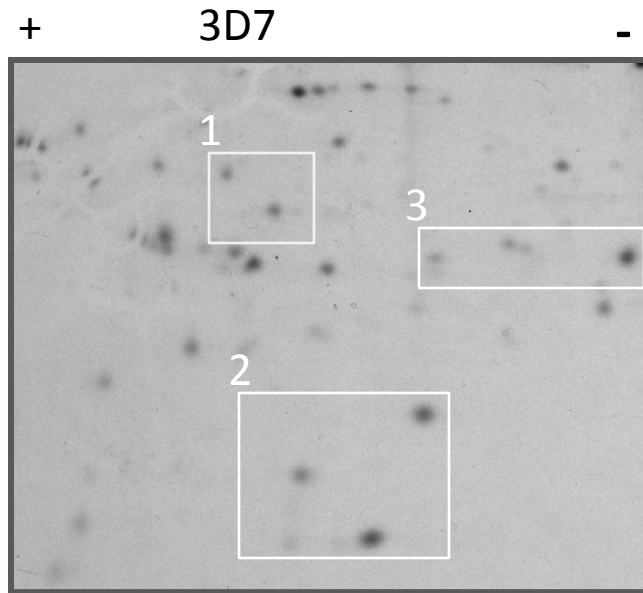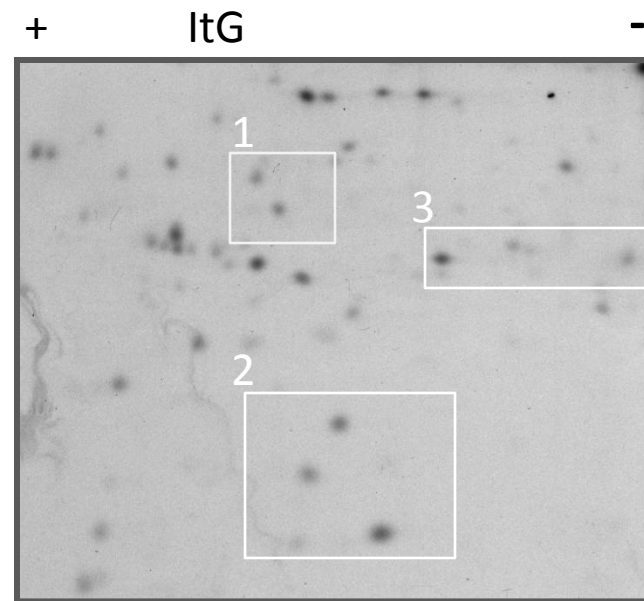

|   | 3D7 | ItG |
|---|-----|-----|
| 1 |     |     |
| 2 |     |     |
| 3 |     |     |

Supplement: Additional file 1 — PI changes of pRBC from different parasite lines. Fluorography of proteins from trophozoite stage pRBC infected with 3D7 and ItG. At 20 hours after invasion, parasites were metabolically labelled with 50 μCi/ml [35S] methionine for 4 hours. Tris-insoluble pellets of pRBC were separated run on pH 4–7 IEF strips followed by 12% SDS-PAGE. Gels were stained with Coomassie blue, dried and exposed to X-ray film. Marked boxes show proteins with at least three fold changes between 3D7 and ItG. Enlarged images of corresponding boxes showing significant changes in the protein profiles. Arrows indicate the relative positions of the spots in different lines. [file 1475-2875-8-105-S1.pdf]

A

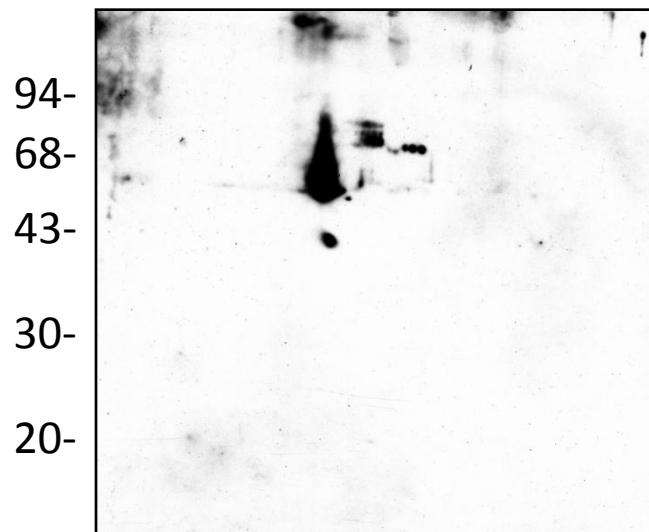

B

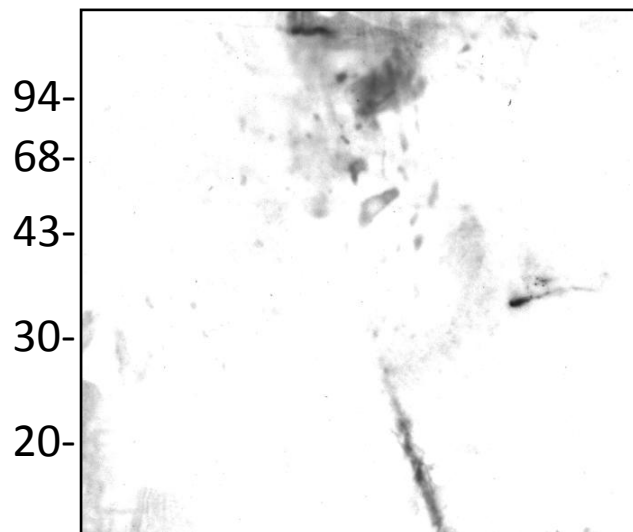

C

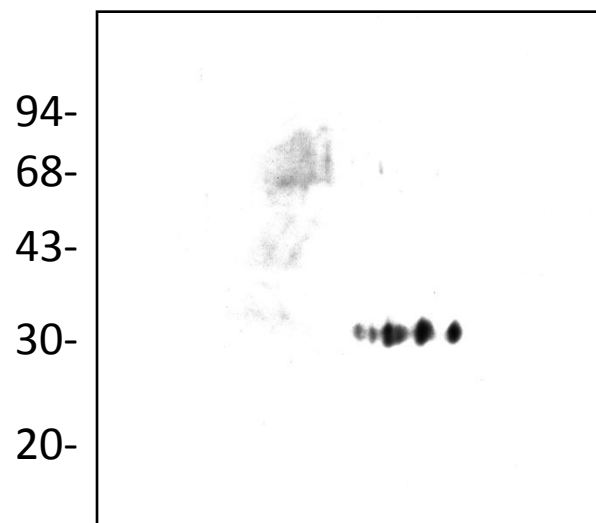

Supplement: Additional file 2 — Immunoblot of normal RBC and secondary antibody control. Immunoblot of normal RBC separated by 2DE (using the same conditions as for Figures 2 &3) and probed with antibodies to phosphorylated serine/threonine (A) or tyrosine (B). Part C is an immunoblot of Tris-insoluble pellet of ItG-infected RBC probed with the secondary antibody only and developed by ECL. [file 1475-2875-8-105-S2.pdf]

A

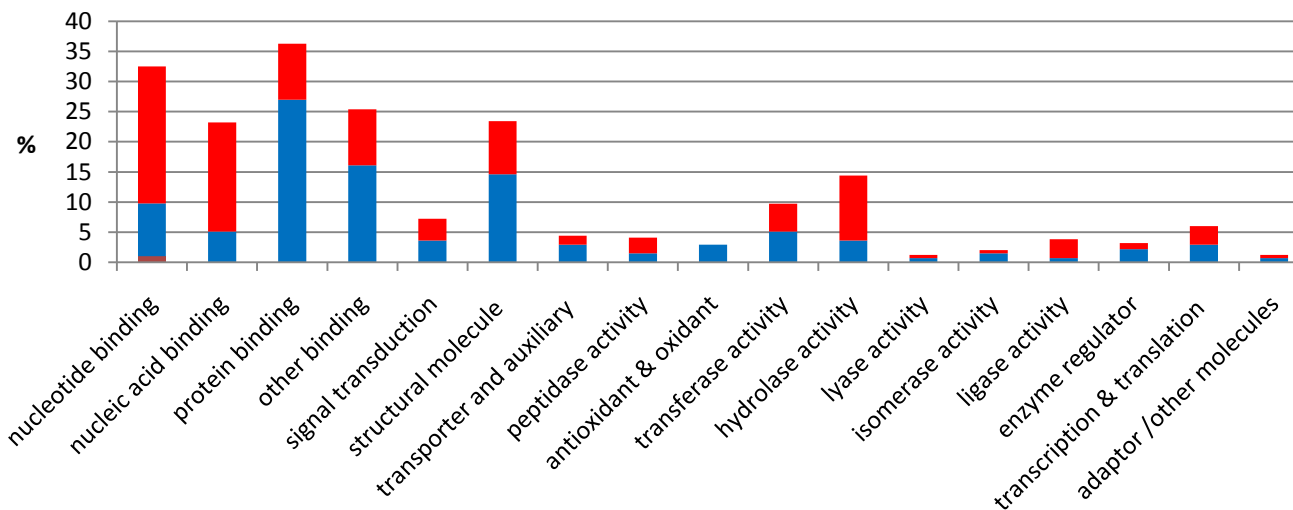

B

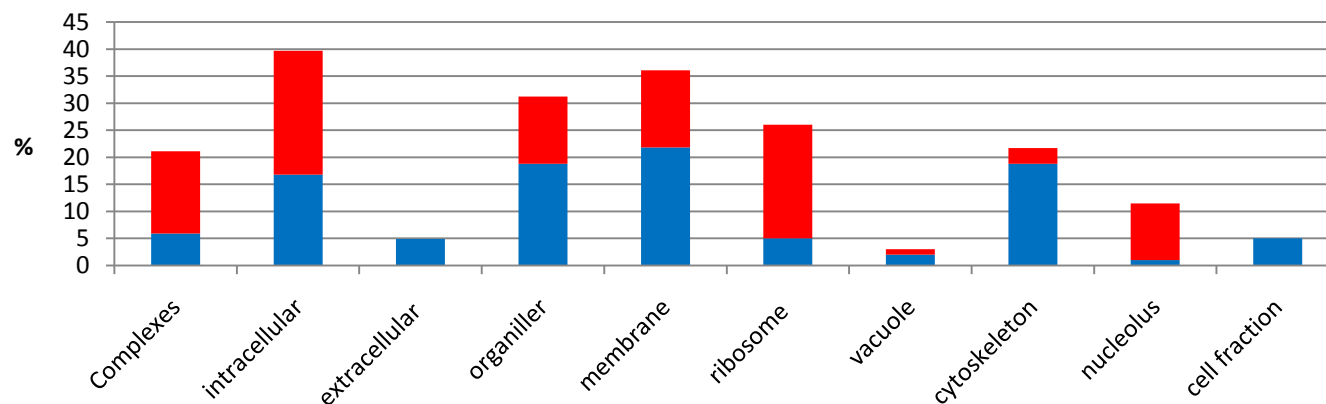

C

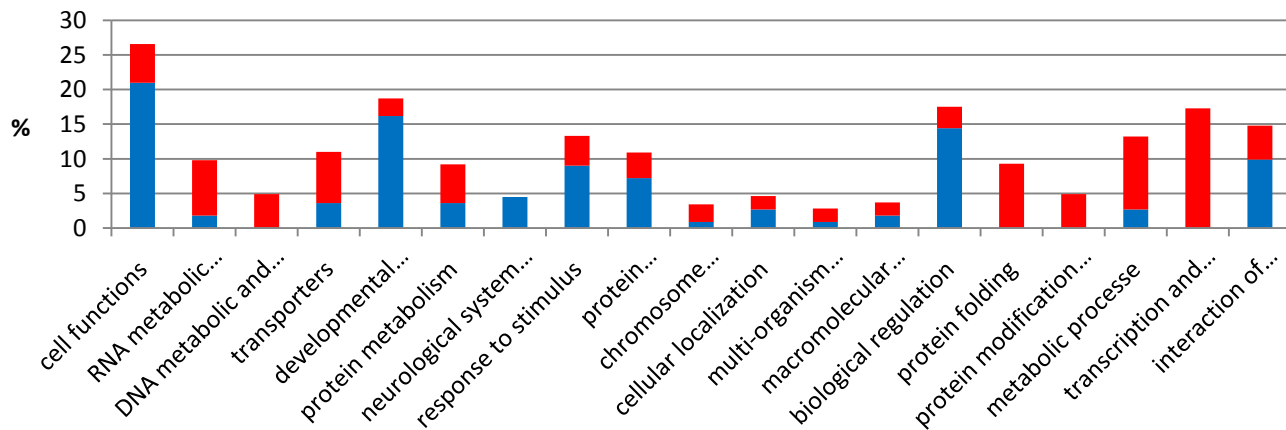

Supplement: Additional file 6 — Gene ontology analysis of serine/threonine and tyrosine phosphorylated proteins. Gene ontology analysis of the serine/threonine and tyrosine phosphorylated proteins. A. Functional categories; B. Cellular components; C. Biological processes. The different colours indicate human (blue) or parasite (red) proteins. [file 1475-2875-8-105-S6.pdf]
