# Supplementary material for: Identification of phosphorylated proteins in erythrocytes infected by the human malaria parasite Plasmodium falciparum
Source: Malar J. 2009 May 18;8:105. doi: 10.1186/1475-2875-8-105 (PMC2696463; doi:10.1186/1475-2875-8-105)
Supplement: Additional file 3 — Plasmodium falciparum phosphorylated proteins from ItG infected erythrocytes. Phosphorylated proteins purified/enriched by affinity chromatography techniques were separated by 1D SDS-PAGE and identified by nano-flow LC/MS/MS. Additional file 4 contains P. falciparum proteins from ItG-pRBC identified using searches including the phosphorylation modifications. [file 1475-2875-8-105-S3.doc]

Supplementary Table 1 - *Plasmodium falciparum* phosphorylated proteins from ItG infected erythrocytes

| Accession number | **Protein Name** | Mol Wt | **PI** | **Score** | Phosphorylation  Details* |
| --- | --- | --- | --- | --- | --- |
| PFE1605w | DNAJ protein | 61390 | 9.34 | 847 | ST4, Y2 |
| PFI1445w | High molecular weight rhoptry protein-2 | 163587 | 8.44 | 802 | ST2, Y1 |
| [PF10_0159](http://www.genedb.org/genedb/Search?name=PF10_0159&organism=malaria&desc=yes&wildcard=yes) | Glycophorin-binding protein (GBP-130) | 90077 | 5.08 | 779 | ST2 |
| [A25526](http://138.253.24.18/mascot/cgi/protein_view.pl?file=../data/20080603/F003607.dat&hit=A25526&px=1&ave_thresh=34&_sigthreshold=0.05&_server_mudpit_switch=1e-009) | ring-infected erythrocyte surface antigen precursor | 125229 | 4.46 | 609 | ST1, Y1 |
| [PF10_0115](http://www.genedb.org/genedb/Search?name=PF10_0115&organism=malaria&desc=yes&wildcard=yes) | QF122 antigen | 132002 | 9.19 | 456 | ST12 |
| [PF08_0054](http://www.plasmodb.org/plasmo/showRecord.do?name=GeneRecordClasses.GeneRecordClass&project_id=&primary_key=PF08_0054) | Heat shock 70 kDa protein (HSP74) | 74754 | 5.51 | 444 | ST3 |
| PFF1185w | iswi protein homologue | 319471 | 6.59 | 379 | ST12, Y1 |
| PFL0100c | ATP dependent RNA helicase (adenosinetriphosphatase) | 122429 | 5.53 | 435 | ST6, Y1 |
| [PF13_0348](http://www.genedb.org/genedb/Search?name=PF13_0348&organism=malaria&desc=yes&wildcard=yes) | rhoptry protein, putative | 15133 | 4.97 | 432 | ST3 |
| [BAG09337](http://www.ncbi.nlm.nih.gov/entrez/viewer.fcgi?db=protein&id=167963114) | RhopH1/Clag3.2 | 167932 | 6.49 | 369 | ST12, Y1 |
| [PFB0935w](http://www.genedb.org/genedb/Search?organism=malaria&name=PFB0935w&isid=true) | cytoadherence linked asexual protein 2 | 171162 | 7.60 | 364 | ST11, Y1 |
| [PF13_0305](http://www.genedb.org/genedb/Search?name=PF13_0305&organism=malaria&desc=yes&wildcard=yes) | Elongation factor 1-alpha (EF-1-alpha) | 49238 | 9.27 | 392 | ST2, Y1 |
| PF13_0214 | elongation factor 1-gamma | 50491 | 8.92 | 89 | ST1 |
| [PFI1445w](http://www.plasmodb.org/plasmo/showRecord.do?name=GeneRecordClasses.GeneRecordClass&project_id=&primary_key=PFI1445w) | high molecular weight rhoptry protein | 163587 | 8.47 | 342 | ST3, Y1 |
| PF14_0393 | structure specific recognition protein, | 58973 | 4.56 | 326 | ST2, Y2 |
| PFF1365c | HECT-domain (ubiquitin-transferase) | 1216945 | 8.19 | 325 | ST5, Y5 |
| [MAL7P1.38](http://www.genedb.org/genedb/Search?name=MAL7P1.38&organism=malaria&desc=yes&wildcard=yes) | regulator of chromosome condensation protein | 79931 | 6.47 | 323 | ST5, Y1 |
| [Q25732_PLAFA](http://138.253.24.18/mascot/cgi/protein_view.pl?file=../data/20080604/F003627.dat&hit=Q25732_PLAFA&px=1&ave_thresh=34&_sigthreshold=0.05&_server_mudpit_switch=1e-009) | Chromodomain protein | 30999 | 9.48 | 323 | ST5 |
| XP_001351065.1 | RESA-like protein, | 36224 | 8.75 | 321 | ST3 |
| PFL0055c | Protein with DNAJ domain | 108185 | 7.04 | 321 | ST1, Y1 |
| [PF11_0313](http://www.plasmodb.org/plasmo/showRecord.do?name=GeneRecordClasses.GeneRecordClass&project_id=&primary_key=PF11_0313) | ribosomal phosphoprotein P0 | 35002 | 6.28 | 288 | ST1, Y1 |
| XP_001351567 | mature parasite-infected erythrocyte surface antigen | 168289 | 4.45 | 267 | ST1 |
| PFI0875w | 78KDa glucose-regulated protein | 72845 | 5.18 | 255 | ST1 |
| [BAG09313](http://www.ncbi.nlm.nih.gov/entrez/viewer.fcgi?db=protein&id=167963325) | RhopH1/Clag3.1 | 169640 | 6.55 | 235 | ST6, Y2 |
| [PFE0675c](http://www.genedb.org/genedb/Search?name=PFE0675c&organism=malaria&desc=yes&wildcard=yes) | deoxyribodipyrimidine photolyase | 130885 | 9.22 | 189 | ST3 |
| [PF14_0316](http://www.plasmodb.org/plasmo/showRecord.do?name=GeneRecordClasses.GeneRecordClass&project_id=&primary_key=PF14_0316) | DNA topoisomerase 2 | 161727 | 8.10 | 187 | ST4, Y1 |
| PF14_0513 | RNA binding protein | 57307 | 5.43 | 181 | ST3, Y1 |
| XP_745954 | N-ethylmaleimide-sensitive fusion protein | 89820 | 6.31 | 181 | ST3 |
| [PF13_0330](http://www.plasmodb.org/plasmo/showRecord.do?name=GeneRecordClasses.GeneRecordClass&project_id=&primary_key=PF13_0330) | ATP-dependent DNA helicase | 54937 | 5.32 | 172 | ST1 |
| PF14_0265 | peptide chain release factor 1 KINASE | 36438 | 10.23 | 168 | ST2 |
| XP_724565 | DNA-directed RNA polymerase | 280047 | 5.35 | 167 | ST3 |
| [PB001156.01.0](http://plasmodb.org/plasmo/showRecord.do?name=GeneRecordClasses.GeneRecordClass&project_id=PlasmoDB&primary_key=PB001156.01.0) | Phenylalanine- tRNA ligase | 73538 | 5.56 | 154 | Y1 |
| [PF10_0153](http://www.plasmodb.org/plasmo/showRecord.do?name=GeneRecordClasses.GeneRecordClass&project_id=&primary_key=PF10_0153) | 60 kDa heat-shock protein PfHsp60 | 62525 | 7.05 | 147 | ST8, Y1 |
| PFF0250w | RNA binding protein, putative | 86204 | 6.47 | 146 | ST1 |
| PFB0100c | Knob-associated histidine-rich protein precursor | 69791 | 9.24 | 138 | ST1, Y1 |
| [AAD10140](http://138.253.24.18/mascot/cgi/protein_view.pl?file=../data/20080604/F003624.dat&hit=AAD10140&px=1&ave_thresh=34&_sigthreshold=0.05&_server_mudpit_switch=1e-009) | acidic ribosomal phosphoprotein PO | 35032 | 6.62 | 130 | ST2 |
| PFE0870w | transcriptional regulator, putative | 133456 | 4.86 | 127 | ST1, Y1 |
| [Q9TY78_PLAFA](http://138.253.24.18/mascot/cgi/protein_view.pl?file=../data/20080603/F003616.dat&hit=Q9TY78_PLAFA&px=1&ave_thresh=34&_sigthreshold=0.05&_server_mudpit_switch=1e-009) | 96 tR antigen | 6726 | 4.58 | 123 | ST2 |
| Q25730 | Rhoptry associated protein-1 | 90423 | 6.69 | 121 | St2 |
| MAL8P1.69 | 14-3-3 protein homologue | 29856 | 4.96 | 115 | ST1 |
| [PFI0645w](http://www.plasmodb.org/plasmo/showRecord.do?name=GeneRecordClasses.GeneRecordClass&project_id=&primary_key=PFI0645w) | EF-1B | 32121 | 4.94 | 113 | ST2 |
| [Q86BT6_PLAFA](http://138.253.24.18/mascot/cgi/protein_view.pl?file=../data/20080603/F003612.dat&hit=Q86BT6_PLAFA&px=1&ave_thresh=34&_sigthreshold=0.05&_server_mudpit_switch=1e-009) | Phoshoprotein 300 | 31110 | 4.72 | 113 | ST3 |
| PFD1165w  [Q8IFM1_PLAF7](http://138.253.24.18/mascot/cgi/protein_view.pl?file=../data/20080603/F003614.dat&hit=Q8IFM1_PLAF7&px=1&ave_thresh=34&_sigthreshold=0.05&_server_mudpit_switch=1e-009) | protein kinase, conserved in P. falciparum | 75365 | 9.05 | 153 | ST8, Y1 |
| PF13_0304 | elongation factor 1 alpha | 49156 | 9.12 | 103 | ST4, Y1 |
| PF11_0507 | antigen 332 | 621141 | 3.78 | 103 | ST4 |
| PF11_0175 | heat shock protein 101 | 103038 | 9.17 | 103 | ST1 |
| [PF07_0029](http://www.genedb.org/genedb/Search?name=PF07_0029&organism=malaria&desc=yes&wildcard=yes) | heat shock protein 86 | 86770 | 4.91 | 103 | ST2 |
| [A28412](http://138.253.24.18/mascot/cgi/protein_view.pl?file=../data/20080603/F003617.dat&hit=A28412&px=1&ave_thresh=34&_sigthreshold=0.05&_server_mudpit_switch=1e-009) | histidine-rich protein precursor | 69791 | 9.24 | 101 | ST9 |
| [PFL2215w](http://www.plasmodb.org/plasmo/showRecord.do?name=GeneRecordClasses.GeneRecordClass&project_id=&primary_key=PFL2215w) | Actin-1 (Actin I) | 42044 | 5.27 | 99 | ST4 |
| [XP_001351787](http://www.ncbi.nlm.nih.gov/entrez/viewer.fcgi?db=protein&id=124506379) | multidrug resistance protein | 162494 | 8.94 | 95 | ST6, Y1 |
| [PFL1545c](http://www.genedb.org/genedb/Search?name=PFL1545c&organism=malaria&desc=yes&wildcard=yes) | Chaperonin CPN60 | 79738 | 4.91 | 94 | ST2 |
| Q8ID50 | uba52 homologue | 14893 | 9.91 | 94 | ST1 |
| [PFL0585w](http://www.plasmodb.org/plasmo/showRecord.do?name=GeneRecordClasses.GeneRecordClass&project_id=&primary_key=PFL0585w) | PfpUB | 42830 | 7.85 | 94 | ST1 |
| [PFL1170w](http://www.genedb.org/genedb/Search?name=PFL1170w&organism=malaria&desc=yes&wildcard=yes) | polyadenylate-binding protei | 97455 | 8.96 | 93 | ST11 |
| Q8MPL1 | Hypothetical protein Dd2-3.5 | 62259 | 5.50 | 91 | ST4, Y1 |
| [JC2374](http://138.253.24.18/mascot/cgi/protein_view.pl?file=../data/20080604/F003635.dat&hit=JC2374&px=1&ave_thresh=34&_sigthreshold=0.05&_server_mudpit_switch=1e-009) | ras-related nuclear GTP binding protein Ran/TC4 homolog | 24974 | 7.72 | 91 | ST2 |
| PF13_0276 | membrane-associated histidine rich protein 2 | 15849 | 7.4 | 89 | ST1 |
| PFD0685c | chromosome associated protein, | 142163 | 6.48 | 89 | ST11, Y2 |
| Q25762 | P82 (rhoptry-associated protein 1) | 32326 | 4.99 | 88 | ST6 |
| [PFI0875w](http://www.plasmodb.org/plasmo/showRecord.do?name=GeneRecordClasses.GeneRecordClass&project_id=&primary_key=PFI0875w) | heat shock protein 70 | 72429 | 5.11 | 87 | ST2 |
| [Q8IIQ7_PLAF7](http://138.253.24.18/mascot/cgi/protein_view.pl?file=../data/20080603/F003607.dat&hit=Q8IIQ7_PLAF7&px=1&ave_thresh=34&_sigthreshold=0.05&_server_mudpit_switch=1e-009) | Asparagine-rich antigen | 174047 | 9.19 | 84 | ST5, Y2 |
| [Q8IKH8_PLAF7](http://138.253.24.18/mascot/cgi/protein_view.pl?file=../data/20080604/F003635.dat&hit=Q8IKH8_PLAF7&px=1&ave_thresh=34&_sigthreshold=0.05&_server_mudpit_switch=1e-009) | Ribosomal protein S3 | 24823 | 10.20 | 82 | ST1 |
| PFB0695c | ATP-dept. acyl-CoA synthetase (TP) | 103277 | 8.83 | 81 | ST8, Y1 |
| PF10_0084 | Tubulin beta chain | 50238 | 4.69 | 76 | ST3, Y1 |
| [Q9GUX2_PLAFA](http://138.253.24.18/mascot/cgi/protein_view.pl?file=../data/20080604/F003634.dat&hit=Q9GUX2_PLAFA&px=1&ave_thresh=34&_sigthreshold=0.05&_server_mudpit_switch=1e-009) | Heat shock protein DnaJ homologue Pfj4 | 28102 | 9.06 | 76 | ST1 |
| [S41717](http://138.253.24.18/mascot/cgi/protein_view.pl?file=../data/20080529/F003520.dat&hit=S41717&px=1&ave_thresh=34&_sigthreshold=0.05&_server_mudpit_switch=1e-009) | aspartic hemoglobinase | 51656 | 6.72 | 75 | ST6 |
| [Q8IE67_PLAF7](http://138.253.24.18/mascot/cgi/protein_view.pl?file=../data/20080604/F003622.dat&hit=Q8IE67_PLAF7&px=1&ave_thresh=34&_sigthreshold=0.05&_server_mudpit_switch=1e-009) | Phosphoribosylpyrophosphate synthetase (EC 2.7.6.1) | 49865 | 9.39 | 75 | ST2 |
| [Q9GTW3_PLAFA](http://138.253.24.18/mascot/cgi/protein_view.pl?file=../data/20080604/F003624.dat&hit=Q9GTW3_PLAFA&px=1&ave_thresh=34&_sigthreshold=0.05&_server_mudpit_switch=1e-009) | Glutamic acid-rich protein | 80987 | 4.91 | 75 | Y1 |
| PFB0300c | MSP2 | 18162 | 4.77 | 74 | ST3 |
| [A39112](http://138.253.24.18/mascot/cgi/protein_view.pl?file=../data/20080604/F003619.dat&hit=A39112&px=1&ave_thresh=34&_sigthreshold=0.05&_server_mudpit_switch=1e-009) | merozoite 45K surface antigen precursor | 30197 | 5.97 | 74 | ST3 |
| PFL0625c | eukaryotic translation initiation factor | 166586 | 6.38 | 73 | ST2 |
| [PFI0740c](http://www.genedb.org/genedb/Search?name=PFI0740c&organism=malaria&desc=yes&wildcard=yes) | Ubiquitin | 18190 | 8.44 | 70 | ST1 |
| [Q8I608_PLAF7](http://138.253.24.18/mascot/cgi/protein_view.pl?file=../data/20080604/F003620.dat&hit=Q8I608_PLAF7&px=1&ave_thresh=34&_sigthreshold=0.05&_server_mudpit_switch=1e-009) | Nucleosome assembly protein 1 | 42199 | 5.97 | 68 | ST2 |
| [PF11_0507](http://www.plasmodb.org/plasmo/showRecord.do?name=GeneRecordClasses.GeneRecordClass&project_id=&primary_key=PF11_0507) | erythrocyte membrane-associated giant protein antigen 332 | 615257 | 3.77 | 67 | ST6 |
| PFI1730w | cytoadherence linked asexual protein; CLAG9 | 161906 | 8.98 | 67 | ST15, Y1 |
| PFL0210c | eukaryotic initiation factor 5a | 17791 | 5.42 | 67 | ST1 |
| [PB000470.03.0](http://plasmodb.org/plasmo/showRecord.do?name=GeneRecordClasses.GeneRecordClass&project_id=PlasmoDB&primary_key=PB000470.03.0) | octapeptide-repeat antigen | 84463 | 8.2 | 66 | ST2 |
| [S41717](http://138.253.24.18/mascot/cgi/protein_view.pl?file=../data/20080604/F003625.dat&hit=S41717&px=1&ave_thresh=34&_sigthreshold=0.05&_server_mudpit_switch=1e-009) | aspartic hemoglobinase (EC 3.4.23.-) | 51656 | 6.72 | 65 | ST6 |
| PFE1400c | Beta adaptin protein, putative | 102841 | 5.53 | 65 | ST4 |
| PFD0725c | arsenical pump-driving ATPase | 43803 | 5.05 | 64 | ST2 |
| [Q8IED8_PLAF7](http://138.253.24.18/mascot/cgi/protein_view.pl?file=../data/20080604/F003640.dat&hit=Q8IED8_PLAF7&px=1&ave_thresh=34&_sigthreshold=0.05&_server_mudpit_switch=1e-009) | 40S ribosomal protein S15 | 17086 | 10.22 | 63 | ST3 |
| [Q8IDS6_PLAF7](http://138.253.24.18/mascot/cgi/protein_view.pl?file=../data/20080604/F003637.dat&hit=Q8IDS6_PLAF7&px=1&ave_thresh=34&_sigthreshold=0.05&_server_mudpit_switch=1e-009) | 60S ribosomal subunit protein L18 | 21847 | 10.62 | 62 | ST1, Y1 |
| [PF10_0077](http://www.genedb.org/genedb/Search?name=PF10_0077&organism=malaria&desc=yes&wildcard=yes) | eukaryotic translation initiation factor 3 subunit | 84517 | 8.26 | 62 | ST1 |
| Q8I608 | Nucleosome assembly protein 1 | 42199 | 4.90 | 62 | ST3 |
| [Q8ILR7_PLAF7](http://138.253.24.18/mascot/cgi/protein_view.pl?file=../data/20080603/F003617.dat&hit=Q8ILR7_PLAF7&px=1&ave_thresh=34&_sigthreshold=0.05&_server_mudpit_switch=1e-009) | DNA replication licensing factor MCM2 | 112239 | 5.50 | 57 | ST4 |
| [Q8I512_PLAF7](http://138.253.24.18/mascot/cgi/protein_view.pl?file=../data/20080604/F003625.dat&hit=Q8I512_PLAF7&px=1&ave_thresh=34&_sigthreshold=0.05&_server_mudpit_switch=1e-009) | Replication factor c subunit 4 | 38127 | 6.95 | 57 | ST2 Y2 |
| PFI0880c | serine/threonine protein phosphatase | 44605 | 8.88 | 56 | ST2 |
| AAC05220 | variant-specific surface protein | 247350 | 6.25 | 56 | ST10, Y1 |
| [T14602](http://138.253.24.18/mascot/cgi/protein_view.pl?file=../data/20080603/F003615.dat&hit=T14602&px=1&ave_thresh=34&_sigthreshold=0.05&_server_mudpit_switch=1e-009) | variant-specific surface protein | 247350 | 6.25 | 56 | ST12, Y1 |
| AY027491 | Tryptophan/threonine rich antigen | 80523 | 5.96 | 55 | ST1 |
| [PF11_0313](http://www.plasmodb.org/plasmo/showRecord.do?name=GeneRecordClasses.GeneRecordClass&project_id=&primary_key=PF11_0313) | Ribosomal phosphoprotein P0 | 35002 | 6.28 | 54 | ST2 |
| [T28634](http://138.253.24.18/mascot/cgi/protein_view.pl?file=../data/20080603/F003609.dat&hit=T28634&px=1&ave_thresh=34&_sigthreshold=0.05&_server_mudpit_switch=1e-009) | variant-specific surface protein 7 | 252331 | 5.37 | 53 | ST3 |
| PFE0160c | Ser/Arg-rich splicing factor | 37677 | 9.76 | 51 | ST1,Y1 |
| [PFL2405c](http://www.genedb.org/genedb/Search?name=PFL2405c&organism=malaria&desc=yes&wildcard=yes) | Pfg377 | 377639 | 5.71 | 49 | ST6, Y1 |
| [CDPK1_PLAF7](http://138.253.24.18/mascot/cgi/protein_view.pl?file=../data/20080603/F003617.dat&hit=CDPK1_PLAF7&px=1&ave_thresh=34&_sigthreshold=0.05&_server_mudpit_switch=1e-009) | Calcium-dependent protein kinase 1 | 61257 | 6.57 | 48 | ST1, Y1 |
| [Q8IFQ4_PLAF7](http://138.253.24.18/mascot/cgi/protein_view.pl?file=../data/20080603/F003617.dat&hit=Q8IFQ4_PLAF7&px=1&ave_thresh=34&_sigthreshold=0.05&_server_mudpit_switch=1e-009) | Erythrocyte membrane protein 1 (PfEMP1) | 252368 | 5.61 | 47 | ST12 |
| XP_001348577 | Protein prenyltransferase alpha subunit | 65438 | 7.51 | 47 | Y1 |
| [Q8IIA4_PLAF7](http://138.253.24.18/mascot/cgi/protein_view.pl?file=../data/20080603/F003615.dat&hit=Q8IIA4_PLAF7&px=1&ave_thresh=34&_sigthreshold=0.05&_server_mudpit_switch=1e-009) | Threonine--tRNA ligase | 120383 | 8.87 | 46 | ST6, Y1 |
| PF14_0664 | biotin carboxylase subunit of acetyl CoA carboxylase | 393489 | 7.67 | 46 | ST9, Y1 |
| [Q8IJP9_PLAF7](http://138.253.24.18/mascot/cgi/protein_view.pl?file=../data/20080604/F003629.dat&hit=Q8IJP9_PLAF7&px=1&ave_thresh=34&_sigthreshold=0.05&_server_mudpit_switch=1e-009) | ADA2-like protein | 301949 | 8.79 | 45 | ST4 Y2 |
| PF10_0143 | nucleoporin Nup100/Nsp100 | 300258 | 8.95 | 44 | ST1 |
| [Q8ILL3_PLAF7](http://138.253.24.18/mascot/cgi/protein_view.pl?file=../data/20080604/F003630.dat&hit=Q8ILL3_PLAF7&px=1&ave_thresh=34&_sigthreshold=0.05&_server_mudpit_switch=1e-009) | Ribosomal protein family L5 | 34205 | 9.78 | 44 | ST2 |
| PF14_0675 | reticulocyte binding protein 2 homolog B | 131677 | 8.94 | 43 | ST3, Y1 |
| O97223 | RNA helicase | 21387 | 5.15 | 43 | ST1 |
| [A71609](http://138.253.24.18/mascot/cgi/protein_view.pl?file=../data/20080603/F003609.dat&hit=A71609&px=1&ave_thresh=34&_sigthreshold=0.05&_server_mudpit_switch=1e-009) | probable secreted protein PFB0675w | 150851 | 8.55 | 43 | ST3 |
| [Q8IDI5_PLAF7](http://138.253.24.18/mascot/cgi/protein_view.pl?file=../data/20080604/F003620.dat&hit=Q8IDI5_PLAF7&px=1&ave_thresh=34&_sigthreshold=0.05&_server_mudpit_switch=1e-009) | Ribosomal protein L17 | 23689 | 10.28 | 43 | Y1 |
| Q95W83_PLAFA | Erythrocyte membrane protein 1 | 297372 | 6.33 | 42 | ST4, Y3 |
| Q4AE84 | RhopH1/Clag8 | 166552 | 8.55 | 42 | ST2 |
| AAL12845 | erythrocyte membrane protein 1 | 297372 | 6.33 | 42 | ST 6, Y3 |
| [Q8IJP3_PLAF7](http://138.253.24.18/mascot/cgi/protein_view.pl?file=../data/20080604/F003631.dat&hit=Q8IJP3_PLAF7&px=1&ave_thresh=34&_sigthreshold=0.05&_server_mudpit_switch=1e-009) | Cysteine--tRNA ligase | 69665 | 6.83 | 42 | ST3 |
| PF14_0655 | RNA helicase-1, putative | 45624 | 5.48 | 41 | ST1 |
| [Q8IAT7_PLAF7](http://138.253.24.18/mascot/cgi/protein_view.pl?file=../data/20080528/F003490.dat&hit=Q8IAT7_PLAF7&px=1&ave_thresh=34&_sigthreshold=0.05&_server_mudpit_switch=1e-009) | ABC transporter | 112227 | 9.54 | 41 | St1 Y1 |
| [Q8IDD4_PLAF7](http://138.253.24.18/mascot/cgi/protein_view.pl?file=../data/20080529/F003521.dat&hit=Q8IDD4_PLAF7&px=1&ave_thresh=34&_sigthreshold=0.05&_server_mudpit_switch=1e-009) | Ser/Thr protein kinase (EC 2.7.1.37) | 478900 | 8.99 | 41 | ST25, Y5 |
| [Q8ID22_PLAF7](http://138.253.24.18/mascot/cgi/protein_view.pl?file=../data/20080603/F003617.dat&hit=Q8ID22_PLAF7&px=1&ave_thresh=34&_sigthreshold=0.05&_server_mudpit_switch=1e-009) | DNA repair endonuclease | 205602 | 7.66 | 41 | ST4, Y1 |
| [Q8I643_PLAF7](http://138.253.24.18/mascot/cgi/protein_view.pl?file=../data/20080604/F003620.dat&hit=Q8I643_PLAF7&px=1&ave_thresh=34&_sigthreshold=0.05&_server_mudpit_switch=1e-009) | Erythrocyte membrane protein 1 (PfEMP1) | 252229 | 5.37 | 41 | ST2, Y1 |
| [Q8I3B6_PLAF7](http://138.253.24.18/mascot/cgi/protein_view.pl?file=../data/20080604/F003636.dat&hit=Q8I3B6_PLAF7&px=1&ave_thresh=34&_sigthreshold=0.05&_server_mudpit_switch=1e-009) | Ras family GTP-ase | 21127 | 5.02 | 40 | ST2 |
| [Q8I4T3_PLAF7](http://138.253.24.18/mascot/cgi/protein_view.pl?file=../data/20080527/F003462.dat&hit=Q8I4T3_PLAF7&px=1&ave_thresh=34&_sigthreshold=0.05&_server_mudpit_switch=1e-009) | PFG377 | 377665 | 5.71 | 39 | ST 7 |
| [Q8I2Q4_PLAF7](http://138.253.24.18/mascot/cgi/protein_view.pl?file=../data/20080529/F003528.dat&hit=Q8I2Q4_PLAF7&px=1&ave_thresh=34&_sigthreshold=0.05&_server_mudpit_switch=1e-009) | Serine/threonine protein phosphatase | 54961 | 4.98 | 39 | ST4, Y1 |
| [Q8IL84_PLAF7](http://138.253.24.18/mascot/cgi/protein_view.pl?file=../data/20080603/F003616.dat&hit=Q8IL84_PLAF7&px=1&ave_thresh=34&_sigthreshold=0.05&_server_mudpit_switch=1e-009) | Metacaspase-like protein | 227743 | 9.22 | 39 | ST4, Y1 |
| [Q8I1T2_PLAF7](http://138.253.24.18/mascot/cgi/protein_view.pl?file=../data/20080603/F003616.dat&hit=Q8I1T2_PLAF7&px=1&ave_thresh=34&_sigthreshold=0.05&_server_mudpit_switch=1e-009) | Nuclear cap-binding protein | 28157 | 9.66 | 38 | ST1 |
| [Q8IFQ6_PLAF7](http://138.253.24.18/mascot/cgi/protein_view.pl?file=../data/20080603/F003616.dat&hit=Q8IFQ6_PLAF7&px=1&ave_thresh=34&_sigthreshold=0.05&_server_mudpit_switch=1e-009) | PfEMP1 | 251162 | 5.37 | 38 | ST7 |
| [Q8IC01_PLAF7](http://138.253.24.18/mascot/cgi/protein_view.pl?file=../data/20080529/F003526.dat&hit=Q8IC01_PLAF7&px=1&ave_thresh=34&_sigthreshold=0.05&_server_mudpit_switch=1e-009) | Cg4 protein | 101042 | 5.54 | 37 | ST1 |
| [Q8II80_PLAF7](http://138.253.24.18/mascot/cgi/protein_view.pl?file=../data/20080604/F003624.dat&hit=Q8II80_PLAF7&px=1&ave_thresh=34&_sigthreshold=0.05&_server_mudpit_switch=1e-009) | ATP-dependent phosphofructokinase | 185789 | 8.55 | 37 | ST1, Y2 |
| [T18378](http://138.253.24.18/mascot/cgi/protein_view.pl?file=../data/20080529/F003534.dat&hit=T18378&px=1&ave_thresh=34&_sigthreshold=0.05&_server_mudpit_switch=1e-009) | variant-specific surface protein 1 | 341697 | 5.87 | 37 | ST8 |
| MAL13P1.237 | hypothetical protein | 42475 | 7.14 | 448 | ST1 |
| PFL0050c | hypothetical protein | 77525 | 4.4 | 436 | ST2, Y1 |
| PF14_0434 | hypothetical protein | 41909 | 9.38 | 421 | ST8 |
| MAL8P1.95 | Hypothetical protein MAL8P1.95 | 37933 | 4.13 | 375 | ST2 |
| [T18418](http://138.253.24.18/mascot/cgi/protein_view.pl?file=../data/20080527/F003459.dat&hit=T18418&px=1&ave_thresh=34&_sigthreshold=0.05&_server_mudpit_switch=1e-009) | hypothetical protein C0120w | 167990 | 6.75 | 370 | ST3 |
| [Q8IKJ1_PLAF7](http://138.253.24.18/mascot/cgi/protein_view.pl?file=../data/20080603/F003607.dat&hit=Q8IKJ1_PLAF7&px=1&ave_thresh=34&_sigthreshold=0.05&_server_mudpit_switch=1e-009) | Hypothetical protein | 113531 | 4.49 | 344 | ST2 |
| [Q8IBY8_PLAF7](http://138.253.24.18/mascot/cgi/protein_view.pl?file=../data/20080527/F003464.dat&hit=Q8IBY8_PLAF7&px=1&ave_thresh=34&_sigthreshold=0.05&_server_mudpit_switch=1e-009) | Hypothetical protein PF07_0042 | 352195 | 5.67 | 341 | ST2, Y2 |
| PF07_0008 | Hypothetical protein PF07_0008 | 27713 | 8.55 | 333 | ST11, Y1 |
| [Q8III3_PLAF7](http://138.253.24.18/mascot/cgi/protein_view.pl?file=../data/20080603/F003612.dat&hit=Q8III3_PLAF7&px=1&ave_thresh=34&_sigthreshold=0.05&_server_mudpit_switch=1e-009) | Hypothetical protein | 69803 | 5.63 | 315 | ST2 |
| PFE1600w | hypothetical protein | 62720 | 4.93 | 289 | ST1,Y1 |
| [Q8IKA6_PLAF7](http://138.253.24.18/mascot/cgi/protein_view.pl?file=../data/20080527/F003470.dat&hit=Q8IKA6_PLAF7&px=1&ave_thresh=35&_sigthreshold=0.05&_server_mudpit_switch=1e-009) | Hypothetical protein | 52593 | 5.21 | 251 | ST1 |
| [Q8IJG6_PLAF7](http://138.253.24.18/mascot/cgi/protein_view.pl?file=../data/20080603/F003607.dat&hit=Q8IJG6_PLAF7&px=1&ave_thresh=34&_sigthreshold=0.05&_server_mudpit_switch=1e-009) | hypothetical protein | 382729 | 5.96 | 217 | ST4 |
| PF08_0137 | hypothetical protein PF08_0137 | 147208 | 5.76 | 210 | ST4, Y2 |
| [Q6LFP7_PLAF7](http://138.253.24.18/mascot/cgi/protein_view.pl?file=../data/20080604/F003640.dat&hit=Q6LFP7_PLAF7&px=1&ave_thresh=34&_sigthreshold=0.05&_server_mudpit_switch=1e-009) | Hypothetical protein | 14830 | 9.06 | 195 | ST2 |
| PF13_0275 | Hypothetical protein PF13_0275 | 32877 | 9.23 | 141 | ST4 |
| [T18421](http://138.253.24.18/mascot/cgi/protein_view.pl?file=../data/20080603/F003612.dat&hit=T18421&px=1&ave_thresh=34&_sigthreshold=0.05&_server_mudpit_switch=1e-009) | hypothetical protein C0140c | 89820 | 6.31 | 171 | ST5, Y2 |
| MAL7P1.174 | Hypothetical protein MAL7P1.174 | 37984 | 8.89 | 156 | ST3 |
| [T18424](http://138.253.24.18/mascot/cgi/protein_view.pl?file=../data/20080604/F003637.dat&hit=T18424&px=1&ave_thresh=34&_sigthreshold=0.05&_server_mudpit_switch=1e-009) | hypothetical protein C0155c | 19151 | 4.18 | 122 | ST1 |
| [Q8I398_PLAF7](http://138.253.24.18/mascot/cgi/protein_view.pl?file=../data/20080603/F003607.dat&hit=Q8I398_PLAF7&px=1&ave_thresh=34&_sigthreshold=0.05&_server_mudpit_switch=1e-009) | Hypothetical protein PFI0250c | 236361 | 8.44 | 120 | ST4 |
| [Q8IAX8_PLAF7](http://138.253.24.18/mascot/cgi/protein_view.pl?file=../data/20080604/F003631.dat&hit=Q8IAX8_PLAF7&px=1&ave_thresh=34&_sigthreshold=0.05&_server_mudpit_switch=1e-009) | Hypothetical protein PF08_0074 | 27299 | 10.58 | 115 | ST4, Y1 |
| [O96121_PLAF7](http://138.253.24.18/mascot/cgi/protein_view.pl?file=../data/20080603/F003616.dat&hit=O96121_PLAF7&px=1&ave_thresh=34&_sigthreshold=0.05&_server_mudpit_switch=1e-009) | Hypothetical protein PFB0080c | 59008 | 6.20 | 113 | ST2 |
| [Q8IL40_PLAF7](http://138.253.24.18/mascot/cgi/protein_view.pl?file=../data/20080603/F003612.dat&hit=Q8IL40_PLAF7&px=1&ave_thresh=34&_sigthreshold=0.05&_server_mudpit_switch=1e-009) | Hypothetical protein | 67791 | 7.98 | 110 | ST1, Y1 |
| PFD0095c | hypothetical protein | 67974 | 4.47 | 97 | ST2 |
| PF10_0361 | hypothetical protein | 192742 | 9.27 | 89 | ST14 |
| [Q6LFD1_PLAF7](http://138.253.24.18/mascot/cgi/protein_view.pl?file=../data/20080527/F003471.dat&hit=Q6LFD1_PLAF7&px=1&ave_thresh=34&_sigthreshold=0.05&_server_mudpit_switch=1e-009) | Hypothetical protein | 486436 | 5.77 | 86 | ST2, Y1 |
| MAL7P1.171 | Hypothetical protein MAL7P1.171 | 244559 | 6.49 | 87 | ST5, Y1 |
| [Q8IE83_PLAF7](http://138.253.24.18/mascot/cgi/protein_view.pl?file=../data/20080604/F003619.dat&hit=Q8IE83_PLAF7&px=1&ave_thresh=34&_sigthreshold=0.05&_server_mudpit_switch=1e-009) | Hypothetical protein PF13_0131 | 42968 | 8.99 | 80 | ST2 |
| [Q8IDN4_PLAF7](http://138.253.24.18/mascot/cgi/protein_view.pl?file=../data/20080604/F003631.dat&hit=Q8IDN4_PLAF7&px=1&ave_thresh=34&_sigthreshold=0.05&_server_mudpit_switch=1e-009) | Hypothetical protein MAL13P1.233 | 25026 | 7.68 | 80 | ST1 |
| [Q8IJX8_PLAF7](http://138.253.24.18/mascot/cgi/protein_view.pl?file=../data/20080529/F003534.dat&hit=Q8IJX8_PLAF7&px=1&ave_thresh=34&_sigthreshold=0.05&_server_mudpit_switch=1e-009) | Hypothetical protein | 11969 | 9.30 | 71 | ST1 |
| [Q8IEI6_PLAF7](http://138.253.24.18/mascot/cgi/protein_view.pl?file=../data/20080604/F003622.dat&hit=Q8IEI6_PLAF7&px=1&ave_thresh=34&_sigthreshold=0.05&_server_mudpit_switch=1e-009) | Hypothetical protein PF13_0076 | 36681 | 8.83 | 69 | ST2 |
| PF14_0732 | hypothetical protein | 68214 | 6.22 | 61 | ST2 |
| [Q8I2X0_PLAF7](http://138.253.24.18/mascot/cgi/protein_view.pl?file=../data/20080604/F003629.dat&hit=Q8I2X0_PLAF7&px=1&ave_thresh=34&_sigthreshold=0.05&_server_mudpit_switch=1e-009) | Hypothetical protein PFI0895c | 37139 | 6.24 | 54 | ST1 |
| [T18508](http://138.253.24.18/mascot/cgi/protein_view.pl?file=../data/20080527/F003459.dat&hit=T18508&px=1&ave_thresh=34&_sigthreshold=0.05&_server_mudpit_switch=1e-009) | hypothetical protein C0715c | 120781 | 8.28 | 52 | St 10, Y1 |
| [Q8I3F1_PLAF7](http://138.253.24.18/mascot/cgi/protein_view.pl?file=../data/20080604/F003624.dat&hit=Q8I3F1_PLAF7&px=1&ave_thresh=34&_sigthreshold=0.05&_server_mudpit_switch=1e-009) | Hypothetical protein PFE1600w | 60720 | 4.93 | 52 | ST2 |
| [Q8IBR5_PLAF7](http://138.253.24.18/mascot/cgi/protein_view.pl?file=../data/20080603/F003615.dat&hit=Q8IBR5_PLAF7&px=1&ave_thresh=34&_sigthreshold=0.05&_server_mudpit_switch=1e-009) | hypothetical protein MAL7P1.95 | 131962 | 9.27 | 49 | ST5 |
| [Q8IAX8_PLAF7](http://138.253.24.18/mascot/cgi/protein_view.pl?file=../data/20080529/F003529.dat&hit=Q8IAX8_PLAF7&px=1&ave_thresh=34&_sigthreshold=0.05&_server_mudpit_switch=1e-009) | Hypothetical protein PF08_0074 | 27299 | 10.58 | 47 | St2 |
| [Q8I396_PLAF7](http://138.253.24.18/mascot/cgi/protein_view.pl?file=../data/20080604/F003620.dat&hit=Q8I396_PLAF7&px=1&ave_thresh=34&_sigthreshold=0.05&_server_mudpit_switch=1e-009) | Hypothetical protein PFI0260c | 725038 | 6.18 | 46 | ST13, Y4 |
| [C71602](http://138.253.24.18/mascot/cgi/protein_view.pl?file=../data/20080527/F003459.dat&hit=C71602&px=1&ave_thresh=34&_sigthreshold=0.05&_server_mudpit_switch=1e-009) | hypothetical protein PFB0940w | 23559 | 8.18 | 45 | ST1 |
| PF10_0321 | hypothetical protein | 67858 | 9.22 | 46 | Y1 |
| [Q8IL76_PLAF7](http://138.253.24.18/mascot/cgi/protein_view.pl?file=../data/20080603/F003615.dat&hit=Q8IL76_PLAF7&px=1&ave_thresh=34&_sigthreshold=0.05&_server_mudpit_switch=1e-009) | Hypothetical protein | 222686 | 9.29 | 41 | ST3 |
| PF13_0071 | hypothetical protein | 67427 | 6.29 | 40 | ST1 |
| [Q8ILD4_PLAF7](http://138.253.24.18/mascot/cgi/protein_view.pl?file=../data/20080529/F003524.dat&hit=Q8ILD4_PLAF7&px=1&ave_thresh=34&_sigthreshold=0.05&_server_mudpit_switch=1e-009) | Hypothetical protein | 86282 | 6.34 | 40 | ST2, Y2 |
| [Q8IIS4_PLAF7](http://138.253.24.18/mascot/cgi/protein_view.pl?file=../data/20080604/F003625.dat&hit=Q8IIS4_PLAF7&px=1&ave_thresh=34&_sigthreshold=0.05&_server_mudpit_switch=1e-009) | Hypothetical protein | 208286 | 5.63 | 39 | ST7, Y1 |
| PFE1485w | hypothetical protein | 225530 | 6.24 | 89 | ST2 Y1 |
| PF14_0648 | hypothetical protein | 238305 | 9.15 | 65 | Y1 |
| PFD0375w | hypothetical protein | 142869 | 5.3 | 77 | Y1 |

* Phosphorylation details: The phosphorylated proteins were identified because they contained phosphorylated peptides. ST means serine/threonine, Y means tyrosine
