# Supplementary material for: Identification of phosphorylated proteins in erythrocytes infected by the human malaria parasite Plasmodium falciparum
Source: Malar J. 2009 May 18;8:105. doi: 10.1186/1475-2875-8-105 (PMC2696463; doi:10.1186/1475-2875-8-105)
Supplement: Additional file 4 — Host phosphorylated proteins from ItG infected erythrocytes. Phosphorylated proteins purified/enriched by affinity chromatography techniques were separated by 1D SDS-PAGE and identified by nano-flow LC/MS/MS. Additional file 5 contains human phosphorylated proteins from ItG-pRBC identified using searches including the phosphorylation modifications. [file 1475-2875-8-105-S4.doc]

Supplementary Table2 - Host phosphorylated proteins from ItG infected erythrocytes

| Accession number | Protein Name | Mol Wt | PI | Score | Phosphorylation Details* |
| --- | --- | --- | --- | --- | --- |
| [M28880](http://www.ncbi.nlm.nih.gov/entrez/viewer.fcgi?val=M28880) | ankyrin | 207138 | 6.15 | 3092 | ST4 |
| AL353894 | Spectrin, alpha, erythrocytic 1 | 281039 | 4.95 | 2811 | ST5 |
| Q59FP5 | Spectrin | 269042 | 5.07 | 1987 | ST6 |
| SPTB1 | Spectrin beta chain | 246894 | 4.96 | 1676 | ST5 |
| AAB47805 | ankyrin 1, erythrocyte splice form 2 | 207138 | 5.72 | 1390 | ST4 |
| [AB209418](http://www.ncbi.nlm.nih.gov/entrez/viewer.fcgi?val=AB209418) | ankyrin 1, erythrocyte splice form 3 | 204518 | 5.72 | 734 | ST10 |
| [SJHUK](http://138.253.24.18/mascot/cgi/protein_view.pl?file=../data/20080529/F003547.dat&hit=SJHUK&px=1&ave_thresh=42&_sigthreshold=0.05&_server_mudpit_switch=1e-009) | ankyrin 1, erythrocyte splice form 1 | 207346 | 5.65 | 828 | ST2 |
| BAD92655 | ankyrin 1, erythrocyte splice form 4 | 208408 | 5.8 | 768 | ST2 |
| [MMHUE4](http://138.253.24.18/mascot/cgi/protein_view.pl?file=../data/20080529/F003547.dat&hit=MMHUE4&px=1&ave_thresh=42&_sigthreshold=0.05&_server_mudpit_switch=1e-009) | erythrocyte membrane protein band 4.1 | 95750 | 5.37 | 591 | ST3 |
| [CAH93400](http://138.253.24.18/mascot/cgi/protein_view.pl?file=../data/20080530/F003561.dat&hit=CAH93400&px=1&ave_thresh=42&_sigthreshold=0.05&_server_mudpit_switch=1e-009) | erythrocyte membrane protein band 4.9 | 45600 | 8.94 | 364 | ST6 |
| NP_003031 | solute carrier family 4, anion exchanger, RBC band 3-like | 101978 | 5.13 | 544 | ST1 |
| Q05764 | Beta-adducin (Erythrocyte adducin subunit beta) | 81129 | 5.67 | 456 | ST3 |
| [S18208](http://138.253.41.239/mascot/cgi/protein_view.pl?file=D:/data/20070301/F006539.dat&hit=S18208&px=1&protscore=455.835714285714&_mudpit=1) | rabphilin-3A-interacting protein | 81260 | 5.67 | 456 | ST3 |
| P14174 | Erythrocyte membrane protein band 4.1 | 66756 | 6.81 | 401 | ST4 Y1 |
| [JC4135](http://138.253.24.18/mascot/cgi/protein_view.pl?file=../data/20080530/F003582.dat&hit=JC4135&px=1&ave_thresh=42&_sigthreshold=0.05&_server_mudpit_switch=1e-009) | chloride conductance regulatory protein, pICln - human | 26370 | 3.97 | 285 | ST3 |
| AAC50223 | dematin 52K chain | 45726 | 9.06 | 275 | ST2 |
| ADDA | Alpha-adducin Erythrocyte adducin | 81304 | 5.60 | 272 | ST2 |
| [M16247](http://www.ncbi.nlm.nih.gov/entrez/viewer.fcgi?val=M16247) | gamma-actin | 26147 | 5.65 | 249 | ST2 |
| Q5VU58 | Tropomyosin 3 | 29019 | 4.72 | 229 | Y1 |
| [M81635](http://www.ncbi.nlm.nih.gov/entrez/viewer.fcgi?val=M81635) | stomatin peptide  Erythrocyte band 7 integral membrane protein | 32548 | 6.85 | 195 | ST3 |
| [BC114450](http://www.ncbi.nlm.nih.gov/entrez/viewer.fcgi?val=BC114450) | EPB41 protein | 83618 | 5.52 | 184 | ST1 |
| Q59F12 | Protein 4.1 variant (Fragment) | 92774 | 5.41 | 184 | ST2, Y1 |
| [AAH56881](http://www.ncbi.nlm.nih.gov/entrez/viewer.fcgi?db=protein&id=34785151) | ADD2 protein | 78576 | 5.19 | 153 | ST3 |
| Q5U1F4 | Hornerin | 283116 | 10.04 | 148 | ST21, Y2 |
| [GFHUC](http://138.253.24.18/mascot/cgi/protein_view.pl?file=../data/20080530/F003583.dat&hit=GFHUC&px=1&ave_thresh=42&_sigthreshold=0.05&_server_mudpit_switch=1e-009) | glycophorin C | 13802 | 4.69 | 148 | ST1 |
| STOM | Erythrocyte band 7 integral membrane protein (Stomatin) | 31751 | 7.90 | 158 | ST1 |
| AAY44382.2 | anti-human-CD3-anti-human-HMWG bi-scFv antibody fragment precursor | 57974 | 6.47 | 139 | ST3 |
| BAB18257 | Anti TNF-alpha antibody light-chain Fab | 23595 | 6.18 | 139 | ST4 |
| [AB064068.1](http://www.ncbi.nlm.nih.gov/entrez/viewer.fcgi?val=AB064068.1) | Ig kappa chain Am37 precursor | 24194 | 7.75 | 139 | ST2 |
| Q502W4 | IGKC protein | 26262 | 8.70 | 139 | ST2 |
| CAA40340 | glycophorin A | 14784 | 5.28 | 139 | ST2 |
| Q6P491 | Hypothetical protein LOC651928 | 26514 | 8.57 | 136 | ST1 |
| [ATHUG](http://138.253.24.18/mascot/cgi/protein_view.pl?file=../data/20080530/F003562.dat&hit=ATHUG&px=1&ave_thresh=42&_sigthreshold=0.05&_server_mudpit_switch=1e-009) | actin gamma 1 | 42108 | 5.31 | 108 | ST1, Y1 |
| Q53FA3 | HSP70-HOM | 70760 | 5.76 | 107 | ST1 |
| [C3HU](http://138.253.24.18/mascot/cgi/protein_view.pl?file=../data/20080529/F003549.dat&hit=C3HU&px=1&ave_thresh=42&_sigthreshold=0.05&_server_mudpit_switch=1e-009) | complement C3 precursor | 188585 | 6.02 | 106 | ST2, Y2 |
| Q6ZN40 | tropomyosin 3 isoform 2 | 29243 | 4.75 | 105 | ST3 |
| [T08796](http://138.253.24.18/mascot/cgi/protein_view.pl?file=../data/20080531/F003589.dat&hit=T08796&px=1&ave_thresh=42&_sigthreshold=0.05&_server_mudpit_switch=1e-009) | tropomyosin | 34980 | 4.81 | 105 | ST2 |
| Q13484 | Ankyrin G119 | 120266 | 8.56 | 98 | ST4 |
| [CAI12854](http://www.ncbi.nlm.nih.gov/entrez/viewer.fcgi?db=protein&id=55958040) | tropomodulin 1 | 40658 | 5.03 | 94 | ST2 |
| Q4JJA0 | Casein kinase 1 | 42196 | 9.55 | 90 | Y1 |
| 1433G | 14-3-3 protein gamma (Protein kinase C inhibitor protein 1) | 28325 | 4.80 | 82 | ST3 |
| PSA7L | Proteasome subunit alpha type 7-like (EC 3.4.25.1) | 28683 | 9.07 | 80 | ST2 |
| Q562Z4 | Actin-like protein | 11548 | 7.11 | 78 | Y1 |
| [LPHUA1](http://138.253.24.18/mascot/cgi/protein_view.pl?file=../data/20080531/F003593.dat&hit=LPHUA1&px=1&ave_thresh=42&_sigthreshold=0.05&_server_mudpit_switch=1e-009) | apolipoprotein A-I precursor | 30759 | 5.56 | 78 | ST1 |
| Q59GP5 | Eukaryotic translation elongation factor 1 alpha 2 variant | 37116 | 7.76 | 76 | ST2 |
| [AK223042](http://www.ncbi.nlm.nih.gov/entrez/viewer.fcgi?val=AK223042) | eukaryotic translation elongation factor 1 alpha 1 | 47207 | 9.19 | 75 | ST4 |
| [1CMYB](http://138.253.24.18/mascot/cgi/protein_view.pl?file=../data/20080604/F003650.dat&hit=1CMYB&px=1&ave_thresh=42&_sigthreshold=0.05&_server_mudpit_switch=1e-009) | hemoglobin beta chain mutant (D99Y) | 16019 | 7.26 | 75 | Y1 |
| Q5RKT7 | Ubiquitin | 18293 | 9.65 | 73 | ST3 |
| Q5T8M7 | Actin, alpha 1, skeletal muscle | 38142 | 5.39 | 71 | ST1, Y1 |
| NP_001014364 | Ifapsoriasin (Filaggrin 2) | 249296 | 8.45 | 71 | ST1, Y1 |
| NP_003280 | Tropomyosin 2 | 33000 | 4.63 | 68 | ST1 |
| Q96H31 | UBC protein | 61600 | 6.83 | 68 | ST1 |
| Q96MH4 | similar to Polyubiquitin 9 | 43622 | 6.01 | 67 | ST2 |
| Q4KKX0 | EPB42 protein | 70096 | 8.68 | 65 | ST1 |
| Q49A90 | RPS27A protein | 10833 | 4.76 | 63 | ST3 |
| NP_002945 | ubiquitin / ribosomal protein S27a | 17964 | 9.68 | 60 | ST2 |
| [CAI19595](http://138.253.24.18/mascot/cgi/protein_view.pl?file=../data/20080530/F003585.dat&hit=CAI19595&px=1&ave_thresh=42&_sigthreshold=0.05&_server_mudpit_switch=1e-009) | AL356504 NID | 435036 | 9.24 | 56 | ST20 |
| AAA49114 | Tropomyosin 4-anaplastic lymphoma kinase fusion protein minor isoform | 16719 | 4,80 | 56 | ST1, Y1 |
| NP_002037 | glyceraldehyde-3-phosphate dehydrogenase | 36053 | 8.57 | 54 | ST2 |
| Q8IWY7 | Tau-tubulin kinase | 183893 | 6.58 | 52 | ST2 |
| [1A42](http://138.253.24.18/mascot/cgi/protein_view.pl?file=../data/20080531/F003589.dat&hit=1A42&px=1&ave_thresh=42&_sigthreshold=0.05&_server_mudpit_switch=1e-009) | carbonic anhydrase ii | 28802 | 6.63 | 52 | ST1 |
| BAB18265 | Anti HBs antibody light-chain Fab (Fragment) | 23460 | 6.48 | 51 | ST3 |
| [O95819](http://www.ncbi.nlm.nih.gov/entrez/viewer.fcgi?db=protein&id=29427585) | mitogen-activated protein kinase kinase 4 | 43815 | 8.28 | 49 | ST1 |
| BC017935 | Alu subfamily SX sequence | 65013 | 11.4 | 48 | ST13, Y1 |
| AAF76859 | chloride ion current inducer protein I(Cln) | 26379 | 4.00 | 68 | ST2 |
| O15018 | PDZ domain-containing protein 2 | 303934 | 7.14 | 46 | ST10 |
| KC1G3 | Casein kinase I isoform gamma-3 (EC 2.7.11.1) | 51755 | 9.29 | 46 | ST2 |
| AAH04261 | PRR7 protein | 28986 | 8.90 | 46 | ST1 |
| BAD96925 | TNF receptor-associated protein 1 variant | 80091 | 8.92 | 43 | ST2 |
| Q3YBA8 | 14-3-3 tau splice variant | 7895 | 9.06 | 45 | ST2 |
| NP_085044.2 | Synaptic proline-rich membrane protein | 31309 | 8.91 | 43 | ST1 |
| BAB61868 | Raichu 404X | 85646 | 6.45 | 43 | ST5 |
| NP_444513.1 | Dermcidin preproprotein | 11391 | 9.00 | 42 | ST1 |
| [S58884](http://138.253.24.18/mascot/cgi/protein_view.pl?file=../data/20080530/F003568.dat&hit=S58884&px=1&ave_thresh=42&_sigthreshold=0.05&_server_mudpit_switch=1e-009) | Ran-binding protein 2 | 362337 | 5.85 | 42 | ST5, Y2 |
| Q96RF1 | HEJ1 | 7522 | 9.00 | 42 | ST1 |
| AAH14944 | SLTM protein | 78153 | 10.8 | 40 | ST3 |
| Q5SQD3 | Tenascin XB | 462830 | 4.90 | 40 | ST2, Y1 |

* Phosphorylation details: The phosphorylated proteins were identified because they contained phosphorylated peptides. ST means serine/threonine, Y means tyrosine
