# Supplementary material for: Identification of phosphorylated proteins in erythrocytes infected by the human malaria parasite Plasmodium falciparum
Source: Malar J. 2009 May 18;8:105. doi: 10.1186/1475-2875-8-105 (PMC2696463; doi:10.1186/1475-2875-8-105)
Supplement: Additional file 5 — Phosphorylated proteins of non-infected erythrocytes purified from phospho-affinity column. Phosphorylated proteins purified/enriched by affinity chromatography techniques were separated by 1D SDS-PAGE and identified by nano-flow LC/MS/MS. Additional file 6 contains human phosphorylated proteins from normal RBC identified using searches including the phosphorylation modifications. [file 1475-2875-8-105-S5.doc]

Supplementary Table 3 - Phosphorylated proteins of non-infected erythrocytes purified from Phospho-affinity column

| Accession number | Protein Name | Mol Wt | PI | Score | Phosphorylation  Details* |
| --- | --- | --- | --- | --- | --- |
| Q5VYL1 | spectrin | 281039 | 4.95 | 6743 | ST2 |
| AAA60579 | beta-spectrin | 247026 | 5.14 | 6110 | ST2 |
| [M16247](http://www.ncbi.nlm.nih.gov/entrez/viewer.fcgi?val=M16247) | gamma-actin | 26147 | 5.65 | 1561 | ST2 |
| [M28880](http://www.ncbi.nlm.nih.gov/entrez/viewer.fcgi?val=M28880) | ankyrin | 207138 | 6.15 | 1489 | ST4 |
| [SJHUK](http://138.253.24.18/mascot/cgi/protein_view.pl?file=../data/20080606/F003682.dat&hit=SJHUK&px=1&ave_thresh=42&_sigthreshold=0.05&_server_mudpit_switch=0.001) | ankyrin 1, erythrocyte splice form 1 | 207346 | 5.65 | 1358 | ST1 |
| Q59F12 | Protein 4.1 variant | 92774 | 5.41 | 1243 | ST3 |
| [AAA63259](http://138.253.24.18/mascot/cgi/protein_view.pl?file=../data/20080606/F003681.dat&hit=AAA63259&px=1&ave_thresh=43&_sigthreshold=0.05&_server_mudpit_switch=0.001) | HUMSPC1 NID | 145795 | 5.06 | 1158 | ST2 |
| [AAB47805](http://138.253.24.18/mascot/cgi/protein_view.pl?file=../data/20080606/F003684.dat&hit=AAB47805&px=1&ave_thresh=42&_sigthreshold=0.05&_server_mudpit_switch=0.001) | HSANKONE42 NID | 204538 | 5.63 | 1138 | ST1 |
| AAH41666 | Adducin 2 (beta) | 73245 | 6.11 | 715 | ST1 |
| [P35611](http://www.ncbi.nlm.nih.gov/entrez/viewer.fcgi?db=protein&id=12644231) | Alpha-adducin | 81304 | 5.60 | 709 | ST4 |
| CAH93400 | hypothetical protein | 45600 | 8.94 | 665 | ST3 |
| [CAH93400](http://138.253.24.18/mascot/cgi/protein_view.pl?file=../data/20080606/F003696.dat&hit=CAH93400&px=1&ave_thresh=42&_sigthreshold=0.05&_server_mudpit_switch=0.001) | CR861337 NID: | 45600 | 8.94 | 663 | ST4 |
| Q96HD4 | Similar to adducin 2 | 30941 | 5.12 | 653 | ST1 |
| [S18208](http://138.253.24.18/mascot/cgi/protein_view.pl?file=../data/20080606/F003683.dat&hit=S18208&px=1&ave_thresh=42&_sigthreshold=0.05&_server_mudpit_switch=0.001) | rabphilin-3A-interacting protein | 81260 | 5.67 | 515 | ST1 |
| [1RQA_D](http://www.ncbi.nlm.nih.gov/entrez/viewer.fcgi?db=protein&id=58176649) | hemoglobin beta subunits are s-nitrosylated | 15622 | 6.76 | 487 | ST4 |
| Q5VU58 | Tropomyosin 3 | 4.72 | 6.17 | 478 | ST2 |
| A27077 | dnaK-type molecular chaperone | 71082 | 5.37 | 438 | ST1 Y1 |
| Q5RE13 | Ras-related protein Rab-1B | 23080 | 5.17 | 436 | Y2 |
| Q5J7V5 | Migration-related gene 1 protein | 52492 | 6.91 | 301 | ST1 |
| NP_997401 | cAMP-dependent protein kinase, alpha-catalytic subunit | 40547 | 8.85 | 262 | ST1 |
| [GFHUC](http://138.253.24.18/mascot/cgi/protein_view.pl?file=../data/20080607/F003701.dat&hit=GFHUC&px=1&ave_thresh=42&_sigthreshold=0.05&_server_mudpit_switch=0.001) | glycophorin C | 13802 | 4.96 | 233 | ST1 |
| [A34655](http://138.253.24.18/mascot/cgi/protein_view.pl?file=../data/20080607/F003706.dat&hit=A34655&px=1&ave_thresh=42&_sigthreshold=0.05&_server_mudpit_switch=0.001) | transforming protein rap1b | 21040 | 5.65 | 220 | ST1 |
| [S34755](http://www.ncbi.nlm.nih.gov/entrez/viewer.fcgi?val=486668) | 14-3-3 protein beta/alpha (Protein kinase C inhibitor protein 1) | 28048 | 4.76 | 193 | Y1 |
| [AAC50223](http://www.ncbi.nlm.nih.gov/entrez/query.fcgi?cmd=search&db=protein&doptcmdl=genpept&tool=mascot&term=AAC50223%5Baccn%5D) | dematin 52K chain | 45726 | 9.06 | 120 | ST2 |
| Q5BKZ7 | Vesicle amine transport protein 1 | 42122 | 5.88 | 115 | ST1 |
| [TVHURA](http://138.253.24.18/mascot/cgi/protein_view.pl?file=../data/20080607/F003705.dat&hit=TVHURA&px=1&ave_thresh=42&_sigthreshold=0.05&_server_mudpit_switch=0.001) | GTP-binding protein N-ras | 21501 | 5.01 | 111 | ST3 Y1 |
| Q5CZH9 | Hypothetical protein DKFZp686I225 | 203674 | 6.23 | 104 | ST4 |
| [AAD42222](http://www.ncbi.nlm.nih.gov/entrez/viewer.fcgi?db=protein&id=5353738) | erythrocyte membrane protein 4.1 | 95750 | 5.42 | 101 | Y1 |
| [S34753](http://138.253.24.18/mascot/cgi/protein_view.pl?file=../data/20080607/F003705.dat&hit=S34753&px=1&ave_thresh=42&_sigthreshold=0.05&_server_mudpit_switch=0.001) | stratifin - human | 27871 | 4.68 | 101 | ST1 |
| Q96HK3 | calmodulin | 17163 | 3.96 | 100 | ST2 |
| [CIHUH](http://138.253.24.18/mascot/cgi/protein_view.pl?file=../data/20080606/F003691.dat&hit=CIHUH&px=1&ave_thresh=42&_sigthreshold=0.05&_server_mudpit_switch=0.001) | calpain (EC 3.4.22.17) | 82465 | 5.49 | 97 | ST2 |
| [AAL12244](http://138.253.24.18/mascot/cgi/protein_view.pl?file=../data/20080607/F003705.dat&hit=AAL12244&px=1&ave_thresh=42&_sigthreshold=0.05&_server_mudpit_switch=0.001) | AY052478 NID | 24780 | 6.97 | 83 | ST3 Y1 |
| Q5VUL2 | RAB3B, member RAS oncogene family | 24970 | 4.85 | 83 | ST1 |
| Q4VJB6 | 14-3-3 protein epsilon isoform transcript variant 1 | 26658 | 4.76 | 83 | ST2 |
| [I57490](http://138.253.24.18/mascot/cgi/protein_view.pl?file=../data/20080607/F003701.dat&hit=I57490&px=1&ave_thresh=42&_sigthreshold=0.05&_server_mudpit_switch=0.001) | guanine nucleotide regulatory protein | 44378 | 8.11 | 81 | ST1 Y1 |
| Q6MZH2 | Hypothetical protein DKFZp686J07132 | 19210 | 9.30 | 74 | ST2 |
| Q4KKX0 | EPB42 protein | 70096 | 8.68 | 73 | ST6 |
| Q53F93 | Palmitoylated membrane protein 1 variant | 53420 | 7.23 | 63 | ST1 |
| Q5T8M7 | Actin, alpha 1, skeletal muscle | 38142 | 5.39 | 63 | ST2 |
| Q5T8M7 | Actin, alpha 1, skeletal muscle | 38142 | 5.39 | 63 | ST2 |
| [S05585](http://138.253.24.18/mascot/cgi/protein_view.pl?file=../data/20080606/F003699.dat&hit=S05585&px=1&ave_thresh=42&_sigthreshold=0.05&_server_mudpit_switch=0.001) | Tropomyosin | 26618 | 4.77 | 61 | ST1 |
| AAD29304 | Kappa 1 immunoglobulin light chain constant region | 11800 | 5.58 | 57 | ST4 |
| [SLC7A4](http://www.ncbi.nlm.nih.gov/sites/?Db=gene&Cmd=retrieve&dopt=full_report&list_uids=6545&log$=databasead&dbfrom=protein) | Ig heavy chain variable region, VH3 family | 12894 | 8.07 | 48 | Y2 |
| Q5RKT7 | Ubiquitin | 18293 | 9.65 | 47 | ST6 |
| Q5TEM5 | Myosin VI | 146293 | 8.78 | 44 | ST1 |
| Q13707 | ACTA2 protein | 37125 | 5.19 | 43 | Y1 |
| Q1L838 | GTBP-ALT | 122267 | 8.50 | 42 | ST3 Y1 |
| [XP_001167799.1](javascript:PopUpMenu2_Set(Menu_prot114583673);) | Alu subfamily SX sequence contamination warning entry | 65013 | 11.4 | 42 | ST4 |

* Phosphorylation details: The phosphorylated proteins were identified because they contained phosphorylated peptides. ST means serine/threonine, Y means tyrosine
